# Feed Composition Differences Resulting from Organic and Conventional Farming Practices Affect Physiological Parameters in Wistar Rats—Results from a Factorial, Two-Generation Dietary Intervention Trial

**DOI:** 10.3390/nu13020377

**Published:** 2021-01-26

**Authors:** Marcin Barański, Dominika Średnicka-Tober, Leonidas Rempelos, Gultakin Hasanaliyeva, Joanna Gromadzka-Ostrowska, Krystyna Skwarło-Sońta, Tomasz Królikowski, Ewa Rembiałkowska, Jana Hajslova, Vera Schulzova, Ismail Cakmak, Levent Ozturk, Ewelina Hallmann, Chris Seal, Per Ole Iversen, Vanessa Vigar, Carlo Leifert

**Affiliations:** 1 Department of Animal Physiology, Faculty of Biology, University of Warsaw, 02-096 Warsaw, Poland; m.baranski@nencki.edu.pl (M.B.); kss25@biol.uw.edu.pl (K.S.-S.); 2 Laboratory of Neurobiology, Nencki Institute of Experimental Biology, Polish Academy of Sciences, Pasteura 3, 02-093 Warsaw, Poland; 3 Institute of Human Nutrition Sciences, Warsaw University of Life Sciences, Nowoursynowska 159c, 02-776 Warsaw, Poland; dominika_srednicka_tober@sggw.edu.pl (D.Ś.-T.); joanna_gromadzka_ostrowska@sggw.edu.pl (J.G.-O.); tomasz_krolikowski@sggw.edu.pl (T.K.); ewa_rembialkowska@sggw.edu.pl (E.R.); ewelina_hallmann@sggw.edu.pl (E.H.); 4 Nafferton Ecological Farming Group, Food and Rural Development, School of Agriculture, Newcastle University, Newcastle upon Tyne, Tyne and Wear NE1 7RU, UK; leonidas.rempelos@ncl.ac.uk (L.R.); gultekin.hasanaliyeva@gmail.com (G.H.); 5 Department of Sustainable Crop and Food Protection, Food and Environmental Sciences, Faculty of Agriculture, Universita Catollica del Sacro Cuore, I-29122 Piacenza, Italy; 6 Department of Food Analysis and Nutrition, Institute of Chemical Technology, UCT Prague, 166 28 Prague, Czech Republic; jana.hajslova@vscht.cz (J.H.); vera.schulzova@vscht.cz (V.S.); 7 Faculty of Engineering and Natural Sciences, Sabanci University, 34956 Istanbul, Turkey; ismail.cakmak@sabanciuniv.edu (I.C.); lozturk@sabanciuniv.edu (L.O.); 8 Human Nutrition Research Centre, Institute of Cellular Medicine, Newcastle upon Tyne NE2 4HH, UK; chris.seal@ncl.ac.uk; 9 Department of Nutrition, Institute of Basic Medical Sciences, University of Oslo, 0372 Oslo, Norway; p.o.iversen@medisin.uio.no; 10 Department of Haematology, Oslo University Hospital, 0424 Oslo, Norway; 11 NatMed, Southern Cross University, Military Rd., Lismore, NSW 2480, Australia; vanessa.vigar@scu.edu.au; 12 SCU Plant Science, Southern Cross University, Military Rd., Lismore, NSW 2480, Australia

**Keywords:** organic feed, conventional feed, pesticides, mineral fertilizer, cadmium, rat physiology, hormonal balance, immune system responsiveness

## Abstract

Recent human cohort studies reported positive associations between organic food consumption and a lower incidence of obesity, cancer, and several other diseases. However, there are very few animal and human dietary intervention studies that provide supporting evidence or a mechanistic understanding of these associations. Here we report results from a two-generation, dietary intervention study with male Wistar rats to identify the effects of feeds made from organic and conventional crops on growth, hormonal, and immune system parameters that are known to affect the risk of a number of chronic, non-communicable diseases in animals and humans. A 2 × 2 factorial design was used to separate the effects of contrasting crop protection methods (use or non-use of synthetic chemical pesticides) and fertilizers (mineral nitrogen, phosphorus and potassium (NPK) fertilizers vs. manure use) applied in conventional and organic crop production. Conventional, pesticide-based crop protection resulted in significantly lower fiber, polyphenol, flavonoid, and lutein, but higher lipid, aldicarb, and diquat concentrations in animal feeds. Conventional, mineral NPK-based fertilization resulted in significantly lower polyphenol, but higher cadmium and protein concentrations in feeds. Feed composition differences resulting from the use of pesticides and/or mineral NPK-fertilizer had a significant effect on feed intake, weight gain, plasma hormone, and immunoglobulin concentrations, and lymphocyte proliferation in both generations of rats and in the second generation also on the body weight at weaning. Results suggest that relatively small changes in dietary intakes of (a) protein, lipids, and fiber, (b) toxic and/or endocrine-disrupting pesticides and metals, and (c) polyphenols and other antioxidants (resulting from pesticide and/or mineral NPK-fertilizer use) had complex and often interactive effects on endocrine, immune systems and growth parameters in rats. However, the physiological responses to contrasting feed composition/intake profiles differed substantially between the first and second generations of rats. This may indicate epigenetic programming and/or the generation of “adaptive” phenotypes and should be investigated further.

## 1. Introduction

Several recent systematic reviews and meta-analyses suggest that there are significant differences in the concentrations of nutritionally relevant compounds between organic and conventional foods [1,2,3]. Organic crops were reported to have higher antioxidant activity and concentrations of a range of antioxidants, while conventional crops had higher concentrations of protein, nitrate, nitrite, and the toxic metal cadmium, and were four times more likely to contain detectable pesticide residues [1]. Organic farming standards prohibit the use of synthetic chemical pesticides, and water-soluble mineral nitrogen and phosphorus fertilizers which are widely used in conventional farming systems, and composition differences between organic and conventional crops were linked to both contrasting crop protection and fertilization regimes [1,4,5,6,7].

Lower phenolic/antioxidant and higher protein concentrations in conventional compared to organic cereal crops were linked to the use of water-soluble, mineral nitrogen fertilizers (NH_4_^+^, NO_3_^−^, urea), which result in higher N-availability (especially early in the growing season) than the green and animal manure-based fertilization regimes used in organic farming systems [1,4,6,7]. The approximately 50% higher cadmium (Cd) levels in conventional cereals have been linked to the use of water-soluble, mineral phosphorus fertilizers, which are known to contain Cd and nickel residues [1,5]. The four times lower incidence of pesticide residues in organic crops is thought to be due to the non-use of chemosynthetic pesticides in organic farming practice [1,8,9]. Pesticide residues are also found in approximately 10% of organic crops, but their concentrations were reported to be 10–100 times lower than in conventional crops, suggesting that they were mainly due to cross-contamination from neighboring conventional farms [1,8,9].

There is evidence from animal and human epidemiological studies that changes in phenolic/antioxidant, pesticide and cadmium intake may affect human health [10,11,12,13,14,15,16,17,18,19,20,21,22,23,24,25,26,27,28,29,30,31,32,33,34,35,36,37,38,39,40,41,42,43,44,45]. Increased dietary intake of antioxidants/(poly)phenols was linked to a reduced prevalence of obesity, type-2 diabetes, and certain cancers [10,11,12,13,14,15], and increased consumption of antioxidant-rich foods was linked to a reduced prevalence of pre-eclampsia [16]. There is also some evidence that high dietary antioxidant intake augments allergic sensitization in young children [17]. In rat models, intake of green tea phenolics was reported to result in a modulation of hormonal balances (e.g., lower blood leptin and insulin levels), and reduced food intake and body weight [18].

In contrast, increased exposure to pesticides (especially those with endocrine-disrupting capacity) was linked to an increased risk of obesity, cancer (including non-Hodgkin’s lymphoma and breast cancer), neurodegenerative diseases, and hypospadias in infants [19,20,21,22,23,24]. Both occupational and dietary exposure to pesticides was also associated with negative effects on reproductive health [21,25,26]. Also, neurotoxic immune system dysregulation, carcinogenic and endocrine-disrupting effects have been demonstrated for many currently used pesticides in animal studies [19,20,21,27]. It should be pointed out that government regulators set maximum residue limits (MRLs) for pesticides in food crops that are orders of magnitude below the concentrations that result in measurable toxicity symptoms in animal studies. The consumption of foods with pesticide residue levels that are below the MRLs is therefore not considered to pose a health risk to consumers by most toxicologists and government regulators [19,28,29,30]. However, this view has recently been challenged for many of the currently used pesticides especially those linked to endocrine and immune system disrupting and neurological effects [19,24,25,26,30,31,32,33,34].

Cd is a nephrotoxin that is absorbed into the body from dietary sources and smoking of tobacco. P-fertilizer (which can contain more than 9 g Cd/ton of P_2_O_5_) is the main agronomic factor resulting in increased Cd-levels in both food crops and tobacco [5,35,36]. There is increasing evidence that chronic exposure to concentrations as low as the current tolerable weekly intake (TWI) (7 µg Cd/kg body weight) set by the FAO/WHO may affect reproductive outcomes and increase the risk of malignancy, including breast-, prostate- and colorectal cancer [35]. Cd has also been described as an endocrine disruptor affecting reproduction in humans [37], as well as mimicking estrogenic effects in the breast tissue of rats [35], and very low Cd concentrations were reported to modulate the in vitro immune response of human lymphocytes and peripheral blood mononuclear cells [38,39]. Associations between heavy metal excretion and hormonal and immunological factors in women with repeated miscarriages have also been reported [40]. Furthermore, there is evidence for additive/synergistic and antagonistic interactions between pesticides, Cd, and/or antioxidants with respect to toxicity, hormonal balances, immune system function, and/or health-related physiological markers [24,41,42,43,44,45].

It is important to recognize that observational studies have linked increased antioxidant intake and/or reduced pesticide and Cd exposure to a similar range of positive health impacts as high levels of organic food consumption [46,47,48,49,50,51,52,53]. Specifically, organic food consumption was found to be associated with a lower incidence of overweight/obesity [50] and a range of diseases including metabolic syndrome [51], certain cancers (e.g., non-Hodgkin’s lymphoma, post-menopausal breast cancer) [46,52], pre-eclampsia [48], hypospadias [47,49]. However, there are uncertainties related to the self-reporting based dietary assessment methods used in cohort studies and confounding effects of lifestyle differences between organic and conventional food consumers [53,54,55,56,57,58]. For example, organic consumer cohorts were reported to have healthier diets and do more exercise, which could also explain the differences in disease prevalence [56,57,58]. A recent systematic literature review, therefore, concluded that there is an urgent need for controlled clinical trials/dietary intervention studies that confirm and provide a mechanistic understanding of the physiological/health impacts associated with organic food consumption in observational studies [53].

The Nafferton Factorial Systems Comparison (NFSC) trial was established in 2001 to quantify the effects of standard crop protection and fertilization regimes used in the UK organic and conventional farming practice on the yield and nutritional composition of cereal, potato, grain, legume, and field vegetable crops [5,6,7,59,60,61]. Crops produced in the NFSC-trials were previously used to produce feeds for a one-generation dietary intervention study with male Wistar rats [59]. Results indicated that both fertilization and crop protection-related feed composition differences have significant effects on a range of physiological parameters in rats, including hormonal balances and immune system responsiveness [59].

Recent intervention studies with experimental animals and human cohort/epidemiological studies have suggested that maternal nutrition and exposure to endocrine-disrupting chemicals may result in epigenetic programming or “adaptive” phenotypes in subsequent generations [24,31,34,62,63,64]. Single generation studies may therefore not allow an accurate assessment of the impact of organic vs. conventional food consumption on physiological parameters linked to obesity and other diseases. However, there have been, to our knowledge, no multi-generational studies with animal models to test the impact of organic vs. conventional food consumption on physiological markers related to health.

The main objective of the dietary intervention trial reported here was therefore to compare the effect of feed composition differences resulting from organic and conventional crop protection and fertilization regimes on growth, hormone, and immune system parameters in a two-generation study with rats. Assessments focused on growth, endocrine, and immunological parameters that were previously described as markers for the risk of overweight/obesity, metabolic syndrome, and other chronic diseases in animals and humans [31,32,33,34,37,38,39,40,41,64,65,66,67]. Analyses were designed to (a) test the hypothesis that differences in maternal nutrition (e.g., intake of antioxidants and endocrine-disrupting chemicals) may result in epigenetic programming or “adaptive” phenotypes in subsequent generations, and (b) develop a mechanistic understanding of the associations between organic food consumption and lower incidences of obesity and other diseases reported in human cohort studies [46,47,48,49,50,51,52,53].

## 2. Materials and Methods

### 2.1. Crop Production Methods

The four crops (barley, potatoes, carrots, and onions) that were used to produce the compound rat feeds were grown within the NFSC trial at the University of Newcastle’s Nafferton Experimental Farm, Northumberland, United Kingdom (54°59.45′ N, 1°53.89′ W). The NFSC is a long-term trial established in 2001 [5,6] to quantify the effects of, and interactions between, crop protection (pesticide-based protocols used in conventional farming (CP) or crop protection according to organic farming standards without chemosynthetic pesticide use (OP)), and fertility management (mineral fertilizer-based protocols used in conventional farming (CF) or composted manure inputs according to organic farming standards (OF)). This design allows four agricultural systems to be compared: (i) organic (organic crop protection with organic fertilization; OPOF), (ii) low input crop protection (organic crop protection with conventional fertilization; OPCF), (iii) low input fertilization (conventional crop protection with organic fertilization; CPOF), (iv) conventional (conventional crop protection with conventional fertilization; CPCF). The field experiment was based on a factorial split-plot design with four blocks in which two crop protection main plots (12 m × 48 m) were divided into two fertility management subplots (12 m × 24 m) [40,41]. The location of crop protection plots and fertility management subplots within the experiment was randomized, with separation strips between crop protection main plots (10 m) and fertilization subplots (5 m). The cultivation regimes for all crops are described in Table S1.

### 2.2. Experimental Feeds

After harvest crops (barley, potato, carrot, and onion) were transported refrigerated (4 °C) to the Warsaw University of Life Sciences in Poland. All crops were dried to uniform moisture content (11 ± 1%) at the Institute of Agricultural and Food Biotechnology (www.ibprs.pl) using a fluidization method that minimizes losses of bioactive compounds and then processed into pelleted rat feeds. This resulted in 16 experimental pelleted feeds being produced, corresponding to four agricultural systems from four replicate blocks, as previously described [59], with barley accounting for 54.5, potato for 10.2, carrot for 3.92, and onion for 0.95% (*w/w*) of the feed. In order to fulfill the nutritional requirements of rats [68], all four feeds were fortified with 6.8% lactoalbumin (whey powder, Pozmlecz Sp. z o.o., Znin, Poland), 11% casein (sodium caseinate, Hortimex Sp. z o.o., Konin, Poland), 5.8% rapeseed oil (First Pressing Kujawski Oil, ZT Kruszwica SA, Poland), 5.4% mineral supplements and 1.4% of a commercial micronutrient/vitamin supplements (Cargill Sp. z o.o., Kiszkowo, Poland). The mineral supplements consisted of 64% calcium carbonate (Labtar, Sp. z o.o., Tarnów Opolski, Poland), 8% calcium phosphate monohydrate (PPH Standard, Sp. z o.o., Lublin, Poland), 8% NaCl (Solino SA, Inowrocław, Poland) and the micronutrient/vitamin supplement consisted of Ca (30.7%), vitamin A (1.5 × 106 units/kg), vitamin D3 (0.1 × 106 unit/kg), vitamin E (8 g/kg), vitamin K3 (0.3 g/kg), vitamin B1 (0.4 g/kg), vitamin B2 (0.4 g/kg), vitamin B3 (1.5 g/kg), vitamin B6 (0.6 g/kg), vitamin B12 (5 mg/kg), biotin (20 mg/kg), folic acid (100 mg/kg), choline (75 g/kg), Fe (7.5 g/kg), Mn (1 g/kg), Cu (0.8 g/kg), Zn (2.5 g/kg), I (15 mg/kg), Co (15 mg/kg), Se (40 mg/kg), antioxidants (3 g/kg), calcium pantothenate (1 g/kg). Feeds were stored in dry and dark conditions at room temperature and used before the expiry date set by the manufacturer.

### 2.3. Analysis of Rat Feeds

The composition of experimental feeds was analyzed using previously described methods [59], and a more detailed description and reference for the individual analysis methods used is provided in the Supplementary Materials.

### 2.4. Experimental Animals, and Rearing/Housing Systems Used

The animal feeding experiment was carried out using male Wistar rats (strain: Wistar Cmd: WI [WU]) obtained from and kept in the rat breeding house of the Polish Academy of Sciences’ Medical Research Centre (Warsaw, Poland) under conditions of controlled light (L:D 12:12) and temperature (22–23 °C) with ad libitum access to the experimental feeds and fresh water. The study was conducted according to the guidelines of the Declaration of Helsinki and all procedures complied with the Polish regulations for animal experimentation. Ethical approval was obtained from the Fourth Local Ethical Committee on Animal Experimentation in Warsaw (protocol code 15/2007).

Adult female (*n* = 32) and male (*n* = 16) rats were randomly assigned to dietary groups receiving one of the 16 experimental batches of rat feed produced (4 production systems treatments × 4 replicate blocks) for three weeks (see Figure S1 for a flow diagram and description of the experimental design). This approach ensured that both the variation between the (a) four replicate field plots of the same crop management treatment and (b) four crop management treatments (OFOP, OFCP, CFOP, CFCP) was maintained in the animal dietary intervention study. Animals within the same group were then transferred to breeding cages (two females and one male per cage) for reproduction. Pregnant females were separated from males and continued to be fed with the same experimental feeds throughout the pregnancy and lactation period. At the age of three weeks, six randomly selected male pups from each group were placed in individual cages and maintained on the same feed as their parents for another nine weeks (first generation (G1)). At the same time, another two females and one male pup from each dietary group were transferred to separate cages. At their sexual maturity, they were transferred to breeding cages to produce the subsequent second generation of rats. As previously, at the age of three weeks, six male pups randomly selected from each dietary group were experimentally fed for another nine weeks (second generation (G2)). With this experimental design, mothers of both generations of rats were on experimental feeds throughout pregnancy and the suckling period, but mothers of the G1 rats were on standard rat feeds prior to conception, while mothers of the G2 rats were on experimental diets throughout their life.

### 2.5. Rat Growth Assessment

The bodyweight of young rats of both generations was recorded weekly and total weight gain was calculated as a difference between the initial (first after weaning) and the final (on the last day of the experiment) body weight. Daily feed intakes were calculated as a difference between feed provided and feed dropped through the mesh floor of the housing cage. The feed intake records and the feed composition analysis results were used to calculate the daily intakes of specific feed chemical compounds. The feed conversion ratio (FCR) was calculated as a total intake of feed in the experimental period divided by total weight gain.

### 2.6. Sampling for Body Composition, Blood, and Spleen Lymphocyte Analysis

Sampling was performed on 12-week-old rats of both generations after a 12-h fasting period under anesthesia with peritoneal thiopental injection (120 mg/kg of body weight). Fresh whole blood was collected directly from the heart and transferred into polypropylene tubes with heparin and then used for evaluation of hematological parameters. Plasma obtained by centrifugation (15 min, 600× *g*, 4 °C) of blood was stored at −20 °C until analyses of hormonal and immune system parameters. The spleens were dissected aseptically and immediately used for the preparation of in vitro cell cultures. The carcasses were collected and stored at −20 °C for body composition analyses.

### 2.7. Body Composition and Physiological Parameters of Animals

Analyses of all parameters were carried out using previously described methods [59]. Body composition, hematological parameters, and plasma immunoglobulins concentrations were assessed in triplicate, and plasma hormones in duplicate, for each rat from all dietary groups. Splenocyte proliferation was assessed in pooled samples (quadruplicate) for all animals.

In autoclaved and homogenized rat carcasses the percentage content of dry mass, ash, protein, and fat were determined by standard analytical methods [69]. The blood hematological parameters (red blood cell count (RBC), white blood cell number (WBC), packed cell volume (PCV), and hemoglobin content (Hb)) were estimated by standard laboratory methods immediately after the collection of blood. Plasma antioxidant activity/capacity was assessed using the TEAC assay as described previously (59). Insulin, leptin, insulin-like growth factor 1 (IGF-1), corticosterone (Cs), and testosterone (Ts) plasma concentrations were measured using radioimmunoassay (RIA) kits (Linco Research, Inc., St. Charles, MO 63304, USA; www.lincoresearch.com). Growth hormone (GH), immunoglobulin A and G (IgA and IgG) plasma concentrations were assessed using enzyme-linked immunosorbent assay (ELISA) kits (Diagnostic Systems Laboratories, Inc., Webster, TX, USA, www.dslabs.com; Bethyl Laboratories, Inc., Montgomery, TX, USA, www.bethyl.com) [70,71,72]. Spleen leukocytes were cultured in vitro for analysis of their proliferation capacity, both spontaneous and mitogen-stimulated; both concanavalin A (ConA, a T-cell specific mitogen, 0.125 µg/well) and lipopolysaccharide (LPS, a B-cell specific mitogen, 2 µg/well) [46] stimulations were used (Sigma-Aldrich Sp, Poznań, Poland, www.sigmaaldrich.com).

Results were expressed in percentage (body composition), and in ng/mL (insulin, leptin, GH, corticosterone, testosterone) or μg/mL (IGF-1, IgA, IgG) of plasma, and in μmol Trolox-equivalent/mL of plasma (TEAC) as a mean value of 24 animals for each agricultural production system (4 field plot replicates × 6 rats). Results of splenocyte proliferation were expressed in counts per minute (cpm) as a mean value from four pooled samples (4 field plot replicates × 1 pooled sample from 6 rats) for each agricultural production system.

### 2.8. Statistical Analyses

The analyses of variance derived from linear mixed-effects models [73] were carried out using the “nlme” package in the R statistical environment (http://www.r-project.org). The results from both generations of rats were analyzed together in order to test the main effects and interactions between generation (G), crop protection (P), and fertilization (F). An additional assessment was also made for each rat generation separately to test the main effects and interactions between crop protection and fertilization management. The hierarchical nature of the experiment was reflected in the random error structure that was specified as block/crop protection/fertilization [74]. Differences between the four crop management combinations (OFOP, CFOP, OFCP, CFCP) were tested post-hoc using Tukey’s HSD test, based on a mixed-effect model, using the “multcomp” package in R statistical environment [74]. Residual normality was tested using the “qqnorm” function and D’Agostino–Pearson tests in R statistical environment. No data transformations were required. *p*-values smaller than 0.05 were considered significant.

The initial three-factor ANOVA (with rat generation, and both crop protection and fertilization regimes used for feed crop production as factors) identified very highly significant main effects of generation and/or interactions between generation, and fertilization and/or crop protection for a large number of physiological parameters assessed (Table S4). This demonstrated the physiological response to the changes in feed composition resulting from contrasting agronomic practices differed considerably between the first and second generation of rats. Separate two-factor ANOVAs for the two generations of rats were therefore carried out for all rat growth parameters (described in this section), plasma hormone balances (see Section 3.3), immunological parameters (see Section 3.4), and basic physiological parameters (see Section 3.5) assessed in rats.

Correlation analyses were carried out in R statistical environment using “Hmisc” package and visualized using “corrplot” package. RDAs were carried out using the CANOCO software [75], with the importance of intake parameters assessed with automatic forward selection within RDAs, using Monte Carlo permutation tests.

## 3. Results

**Figure 1:**
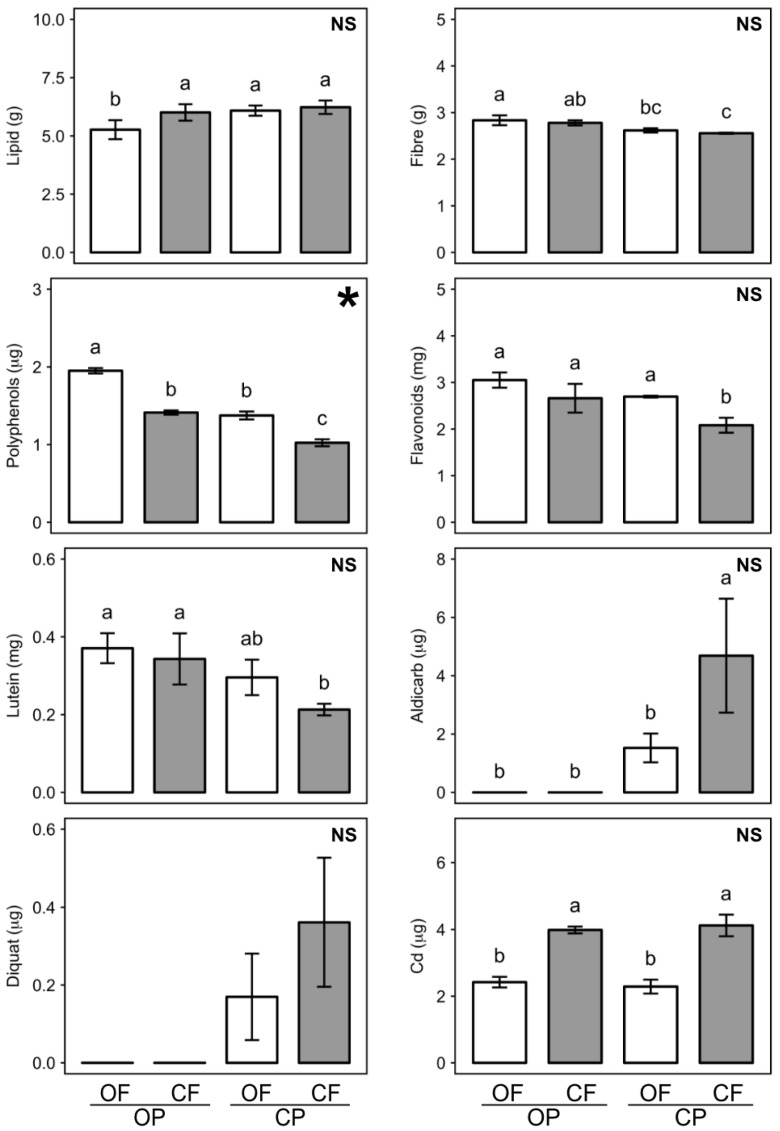
Effects of organic crop protection (OP) or conventional crop protection (CP) and organic fertility management (OF, white bars) or conventional fertility management (CF, shaded bars) on the lipid, fiber, polyphenols, flavonoids, lutein, aldicarb, diquat and cadmium content (per 100 g fresh weight) in experimental feeds. Results shown as means ± SEM of *n* = 4 field replicates, different letters above bars indicate significant difference (*p* < 0.05) determined by Tukey’s HSD test. * two-factor ANOVA detected a significant interaction between crop protection and fertilization regimes (see Table S3).

**Figure 2:**
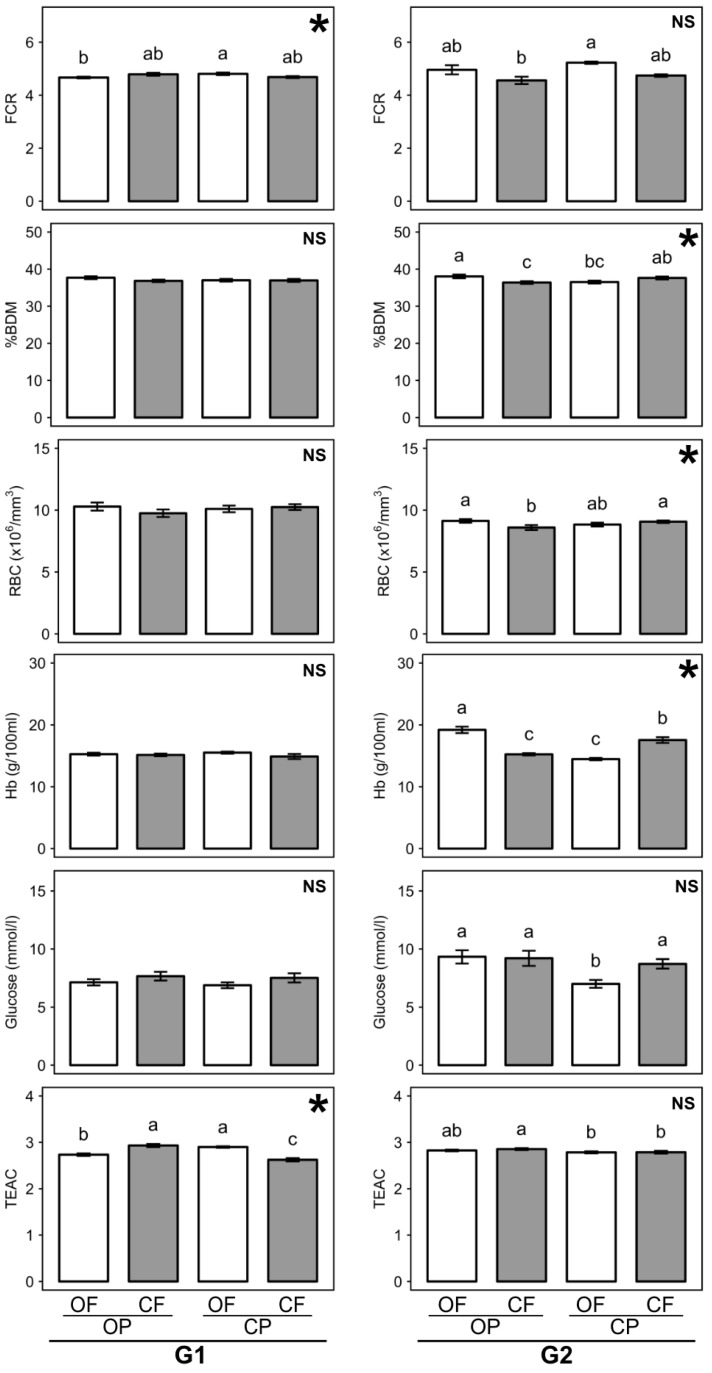
Effects of organic crop protection (OP) or conventional crop protection (CP) and organic fertility management (OF) or conven Table 1. and second (**G2**) generation of rats. Results shown as means ± SEM of *n* = 24 animals, different letters above bars indicate significant difference (*p* ≤ 0.05) determined by Tukey’s HSD test. * two-factor ANOVA detected a significant interaction between crop protection and fertilization regimes.

**Figure 3:**
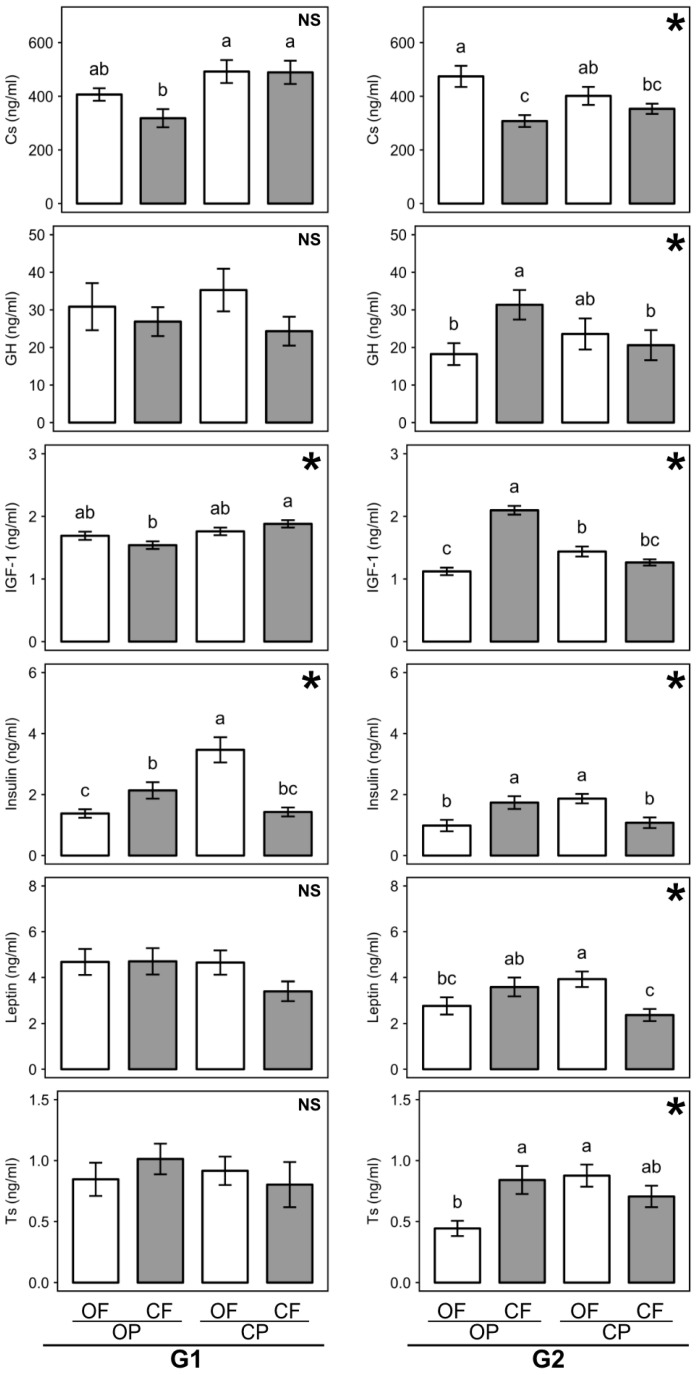
Effects of organic crop protection (OP) or conventional crop protection (CP) and organic fertility management (OF, white bars) or conventional fertility management (CF, shaded bars) on the plasma concentrations of corticosterone (Cs), growth hormone (GH), insulin-like growth factor 1 (IGF-1), insulin, leptin and testosterone (Ts) of the first (**G1**) and second (**G2**) generation of rats. ReScheme 24 animals, different letters above bars indicate significant difference (*p* < 0.05) determined by Tukey’s HSD test. * two-factor ANOVA detected a significant interaction between crop protection and fertilization regimes (see Table S6).

The data presented in Table 1, Tables S2, S3, S5, and S6 are the main effect means from two-factor ANOVA for the crop protection (with and without pesticides) and fertilization (nitrogen, phosphorus and potassium (NPK) -fertilizer vs. manure) treatments, while means shown in Figure 1, Figure 2, Figure 3, Figures S2 and S3 are means obtained for individual feeds (OFOP, CFOP, OFCP, CFCP).

### 3.1. Effect of Agronomic Practices on Feed Composition

The composition of four different compound feeds made from crops (barley, potato, carrot, and onion) produced in 16 experimental plots (4 management protocols × 4 replicate field plots) managed with contrasting crop protection (with and without pesticides) and fertilization (mineral NPK vs. manure as fertilizer) regimes was assessed and compared by two-factor ANOVA (Figure 1; Table S2).

We found significant differences in lipid, fiber, antioxidant (polyphenol, flavonoid, and lutein) and contaminant (aldicarb, diquat, and Cd) profiles between the four different rat feeds (OFOP, CFOP, OFCP, CFCP), but there were no significant differences in protein, ash, β-carotene, N, Cu, Ni, and Pb concentrations (Figure 1, Table S2).

Significant main effects were detected for both crop protection and fertilization regimes used to produce feed crops (Table S2). Specifically, the use of pesticides for crop protection resulted in significantly higher lipid (9%), but lower fiber (8%), flavonoid (16%), lutein (31%), and polyphenolic (29%) concentrations, while compared to manure, the use of mineral NPK-fertilizer resulted in significantly lower polyphenolic (21%), but higher cadmium (72%) concentrations in rat feed (Table S2).

Although a wide range of pesticides was applied to conventionally protect (CP) crops, aldicarb (a carbamate insecticide used in potato and carrots) and diquat (a herbicide used in potato) residues were the only pesticides detected as residues in CP crops (Table S2). The use of NPK-fertilizer resulted in more than two times higher aldicarb and diquat concentrations in feeds made from CP-crops than the use of manure as fertilizer, but the difference was only statistically significant for aldicarb (Figure 1).

A significant interaction (*p* = 0.004) between crop protection and fertilization methods was only detected for the polyphenol concentrations in rat feeds. Pesticide and mineral NPK use had an additive effect with respect to reducing polyphenol concentrations in feeds and this resulted in polyphenol concentrations being 91% higher in feeds made with organic (OFOP) compared to conventionally (CFCP) managed crops (Figure 1). However, it should be pointed out that concentrations of polyphenols, fiber, flavonoid, and lutein all followed the same trend (OFOP > CFOP = OFCP > CFCP) (Figure 1).

Based on these results the composition differences between the four rat feeds used for the dietary intervention study can be summarized as (1) OFOP (=organic feed): low lipid, high fiber, high antioxidant, no pesticide residues, low Cd; (2) OPCF: high lipid, intermediate fiber, intermediate antioxidant, no pesticide residues, high Cd); (3) OFCP: high lipid, intermediate fiber, intermediate antioxidant, low pesticide residues, low Cd); (4) CFCP (=conventional feed): high lipid, low fiber, low antioxidant, high pesticide residues, high Cd).

For the compounds shown in Figure 1, differences in feed composition broadly matched those found when total dietary intakes by G1 and G2 rats were calculated (Figures S2 and S3, Table S3). However, it should be pointed out, that both the use of mineral fertilizer and pesticides for feed crop production resulted in numerically higher feed intakes (Table 1).

As a result, significant effects of fertilization and crop protection regimes were detected for a larger number of feed components when the dietary intakes (Figures S2 and S3; Table S3) instead of concentrations in feed (Figure 1, Table S2) were compared. Notably, when feed intake data were analyzed, protein intake was found to be significantly higher in G1 and G2 rats consuming mineral NPK-fertilized (CFOP, CFCP) compared to manure-fertilized (OFOP, OFCP) feed crops (Figure S2), and Pb intake was found to be significantly (~35%) higher in G1 and G2 rats consuming conventional (CFCP) feed compared to rats consuming OFOP, CFOP, and OFCP feeds, which had a similar daily Pb intake (Figure S3).

### 3.2. Effect of Feed Composition on Rat Growth Parameters

Growth parameters were assessed as potential markers for the risk for obesity and associated diseases. It should be pointed out that the parents of G1 rats were raised on standard rat feeds before mating and were transferred onto one of the four experimental feeds/feed crops only after mating. This means that the parents of the G1 were raised on feeds made from conventionally produced crops.

#### 3.2.1. Initial Body Weight at Weaning

We found a highly significant main effect of generation on the initial body weight at weaning and a significant two-way interaction between generation and fertilization regimes, and generation and crop protection regimes used to produce feed crops (Table S4). The initial body weight at weaning was substantially higher in G2 than G1 rats (Table 1). When data for G1 and G2 rats were analyzed separately by two-factor ANOVA, consumption of contrasting feed crops by the mothers of G1 rats had no significant effect on the weight at weaning of G1 rats. In contrast, G2 rats from mothers consuming pesticide-treated feed crops (CFCP and OFCP) had a significantly higher (~20%) initial body weight at weaning than rats born to mothers consuming non-pesticide treated feed crops (OFOP and CFOP) (Table 1).

#### 3.2.2. Feed Intake

When data for the average daily feed intake in the nine weeks after weaning were analyzed by three-factor ANOVA, a highly significant main effect of generation (feed intake was significantly lower in the G2 than the G1), a significant two-way interaction between generation and fertilization regimes, and a weak trend (*p* = 0.099) towards a three-way significant interaction was detected (Table S4).

When data for G1 and G2 rats were analyzed separately by two-factor ANOVA, no significant differences in feed intake between rats consuming the four experimental feeds could be detected in the G1. In contrast, in G2 rats, consumption of pesticide-treated feed crops (OFCP, CFCP) resulted in a slightly (~5%), but significantly higher feed intake than consumption of untreated feed crops (OFOP, CFOP) (Table 1).

#### 3.2.3. Total Weight Gain

When data for the total weight gain in the nine weeks after weaning were analyzed by three-factor ANOVA, significant main effects of generation (*p* < 0.001) and fertilization (*p* = 0.006), but no significant interactions, were detected (Table S4).

Total weight gains from all four feeds were lower in the G2 than the G1 and overall consumption of mineral NPK fertilized feed crops (CFOP, CFCP) resulted in significantly higher (*p* = 0.006) weight gains than manure fertilized feed crops (OFOP, OFCP) (Table S4). However, when data for G1 and G2 rats were analyzed separately by two-factor ANOVA, a trend (*p* = 0.05) towards a higher total weight gain from mineral NPK fertilized feed crops was only detected in the G1 (Table 1).

#### 3.2.4. Feed Conversion Ratio

Differences in feed conversion ratio (FCR) between the two generations of rats raised on the four different feed crops were relatively small, but some were significant, and the relative differences between feed crops were greater in the G2 than the G1 (Table 1; Figure 2). When data for the feed conversion ratios (FCR) were analyzed by three-factor ANOVA, a significant main effect of generation (*p* = 0.013) and a significant interaction (*p* < 0.001) between generation and the fertilization regimes used to produce feed crops were detected (Table S4). Overall, the FCR was significantly higher (*p* = 0.013) in the G2 than the G1.

However, when data for G1 and G2 rats were analyzed separately by two-factor ANOVA, a significant interaction between fertilization and crop protection regimes was identified in the G1, and a main effect of fertilization in the G2, where consumption of manure-fertilized feed crops (OFOP, OFCP) resulted in a higher FCR than consumption of mineral NPK-fertilized feed crops (CFOP, CFCP) (Table 1). When the interaction between crop protection and fertilization regimes in the G1 (Table 1) was further investigated, the FCR was found to be significantly higher in rats consuming manure fertilized crops treated with pesticides (OFCP-feeds) than rats consuming manure fertilized crops not treated with pesticides (OFOP-feeds). In contrast, the FCR in rats consuming OFCP and CFCP feeds was not significantly different (Figure 2).

### 3.3. Effect of Feed Composition on Rat Body Composition

Rat body composition parameters (body dry matter (%BDM), protein, fat, and ash content) were assessed as potential markers for obesity risk and toxic effects of feed components. No significant differences in body dry matter, fat, and ash content were detected, but three-factor ANOVA detected significant main effects for generation and fertilization regime for body protein concentrations (Tables S4 and S5). Protein content was higher in the G2 than the G1 and consumption of feeds made from NPK fertilized crops (CFOP, CFCP) resulted not only in significantly higher protein intake (Figure S3) but also a higher body protein content (Tables S4 and S6) compared to feeds made from manure fertilized crops (OFOP, OFCP) (Tables S4 and S5). However, when data for G1 and G2 rats were analyzed separately by two-factor ANOVA, only a trend (*p* = 0.06) towards higher body protein content from feeds made from mineral NPK fertilized crops was detected in the G1 (Table S5).

Two-factor ANOVA also identified a significant interaction for the percentage of BDM in the G2 (Figure 2), although differences between treatments were relatively small (Figure 2). Consumption of manure-fertilized feed crops treated with pesticides (OFCP) resulted in a lower percentage of BDM than feeds made from untreated manure fertilized crops (OFOP). In contrast, when feeds made from mineral fertilized crops were compared, consumption of pesticides treated feed crops (CFCP) resulted in a higher percentage of BDM than feeds made from untreated crops (CFOP) (Figure 2).

It is important to point out that no significant differences in body composition could be detected between rats consuming organic (OFOP) and conventional (CFCP) feeds (Figure 2; Table S5).

### 3.4. Effect of Feed Composition on Basic Physiological Parameters in Blood

White and red blood cell (WBC, RBC) count, packed cell volume (PCV), and/or plasma hemoglobin (Hb) levels were assessed as markers of hematopoietic activity, while blood glucose level and plasma antioxidant status (TEAC) were assessed as energy metabolism and anti-oxidative stress capability markers respectively.

Three-factor ANOVA detected highly significant (*p* < 0.001) main effects of generation for five of the six basic physiological parameters assessed in blood nine weeks after weaning (Table S4). RBC and PCV were higher in the G1, while WBCs, Hb, and glucose levels were higher in G2 rats (Figure 2; Table S5). Also, three-way interactions between generation, crop protection, and fertilization regimes used for feed crop production were detected for Hb and TEAC (Table S4). Three-way interactions were further investigated by carrying out separate two-factor ANOVA for G1 and G2 data.

Two factor ANOVA detected no significant differences in Hb levels between G1 rats consuming the four different feeds/feed crops, while in the G2 a significant interaction between fertilization and pesticide regimes used for feed crop production was detected (Figure 2). Consumption of manure-fertilized feed crops treated with pesticides (OFCP) resulted in lower Hb levels than consumption of organic feed crops (OFOP), while conventional feed crops (CFCP) resulted in higher Hb levels compared to untreated, mineral NPK fertilized crops (Figure 2). Also, consumption of organic (OFOP) feed resulted in slightly, but significantly, higher Hb levels than consumption of conventional (CFCP) feeds (Figure 2). The interactions identified for Hb and RBC in G2 rats were similar (Figure 2).

For plasma antioxidant activity (TEAC) a significant interaction between the use of mineral fertilizer and pesticide for feed crop production was detected in the G1 and a significant main effect of crop protection in the G2 (Figure 2; Table S5). In the G1 consumption of manure-fertilized feed crops treated with pesticides (OFCP) resulted in significantly higher, while consumption of pesticide-treated, mineral NPK-fertilized feed crops (CFCP) resulted in significantly lower TEAC levels than consumption of untreated feed crops (OFOP, CFOP) (Figure 2). In the G2 consumption of pesticide-treated feed crops (OFCP, CFCP) resulted in higher plasma TEAC levels than consumption of untreated feed crops (OFOP, CFOP) (Figure 2; Table S5). It should be noted that plasma TEAC levels were positively correlated with dietary polyphenol, flavonoid, and lutein intakes and that the correlation with polyphenols was stronger in the G2 (Figures S7 and S8)

For blood glucose, no significant differences in rats consuming the four different feed crops were detected in the G1, while a significant main effect of crop protection and a trend (*p* = 0.05) towards a significant interaction was detected in the G2 (Figure 2; Table S5). Blood glucose levels were significantly lower in G2 rats consuming pesticide-treated, manure fertilized feed crops (OFCP) than in G2 rats consuming the other three feeds (OFOP, CFOP, CFCP) which resulted in similar blood glucose levels (Figure 2).

It is important to point out that significant differences in basic physiological parameters between rats consuming organic (OFOP) and conventional (CFCP) feed were only detected for TEAC in the G1 and Hb in the G2, which were both higher with organic feed (Figure 2).

### 3.5. Effect of Feed Composition on Plasma Hormone Profiles

Plasma hormone concentrations were assessed in male rats nine weeks after weaning. Three-factor ANOVA identified significant main effects of generation for all hormones except corticosterone (Cs) for which only a trend (*p* = 0.06) was detected (Table S4). Overall, plasma growth hormone ((GH), insulin-like growth factor (IGF-1), insulin, leptin, and testosterone (Ts) concentrations were higher in the G1 than the G2 (Table S6). However, interactions between generation, and fertilization, and/or crop protection were also detected for Cs, IGF-1, insulin, and leptin (Table S4).

Although three-factor ANOVA detected no significant main effects of crop protection, significant main effects of fertilization were detected for Cs and IGF-1 (Table S4) and there were significant interactions between fertilization and crop protection for plasma Cs in the G2 and IGF-1 in both generations (Figure 3; Table S4). Separate two-factor ANOVA tests for data from G1 and G2 rats were therefore carried out to further investigate these interactions.

In the G1, two factor ANOVA found significant differences in blood hormone concentrations between rats raised on the four contrasting feeds for three (Cs, IGF-1, and insulin) of the 6 hormones assessed (Figure 3; Table S6). For all three hormones, significant main effects of crop protection were detected. Cs, IGF-1, and insulin concentrations were significantly higher (by 35, 13, and 40% respectively) in rats consuming pesticide-treated feed crops (OFCP, CFCP) compared to rats consuming untreated feed crops (OFOP, CFOP) (Table S6).

For IGF-1 and insulin, two factor ANOVA also identified significant interactions between crop protection and fertilization (Figure 3; Table S6). Consumption of manure-fertilized, pesticide-treated (OFCP), and untreated (OFOP) feed crops resulted in similar plasma IGF-1 levels, while consumption of mineral NPK-fertilized feed crops treated with pesticides (CFCP) resulted in significantly higher IGF-1 concentrations than consumption of untreated mineral NPK-fertilized feed crops (CFOP) (Figure 3).

In contrast, consumption of pesticide-treated, manure fertilized feed crops (OFCP) resulted in significantly higher insulin levels compared to consumption of untreated, manure fertilized feed crops (OFOP), while consumption of pesticide-treated and untreated, NPK-fertilized feed crops resulted in similar plasma insulin levels (CFOP and CFCP) (Figure 3).

In the G2, two-factor ANOVA detected significant differences in blood hormone concentrations between rats raised on the four contrasting feeds and significant interactions between fertilization and crop protection for all six hormones assessed (Figure 3; Table S6).

The interactions for plasma GH, IGF-1, insulin, leptin, and Ts all had similar trends. Consumption of pesticide-treated, manure-fertilized feed crops (OFCP) resulted in higher GH, IGF-1, insulin, leptin, and Ts levels compared to consumption of untreated, manure-fertilized feed crops (OFOP), although the difference was not significant for GH (Figure 3). In contrast, consumption of pesticide-treated, NPK-fertilized feed crops (CFCP) resulted in lower blood GH, IGF-1, insulin, leptin, and Ts levels compared to consumption of untreated, manure-fertilized feed crops (OFOP), although the difference was not significant for testosterone (Figure 3).

A different trend was detected for plasma Cs levels, with numerically lower values found in rats consuming pesticide-treated (OFCP) compared to untreated (OFOP) manure-fertilized feed crops, but numerically higher Cs levels were found in rats consuming pesticide-treated (CFCP) compared to untreated (CFOP) mineral NPK-fertilized feed crops (Table S6). For Cs, two-factor ANOVA also detected a trend (*p* = 0.06) towards a significant effect of fertilization in the G2, and consumption of OFOP resulted in significantly higher Cs blood levels than consumption of CFOP feeds (Figure 3, Table S6).

It is important to point out that a significant difference between rats consuming organic (OFOP) and conventional feed was only detected for Cs in the G2 (Figure 3).

### 3.6. Effect of Feed Composition Differences on Immune System Parameters

#### 3.6.1. Plasma Immunoglobulin A and G

**Figure 4:**
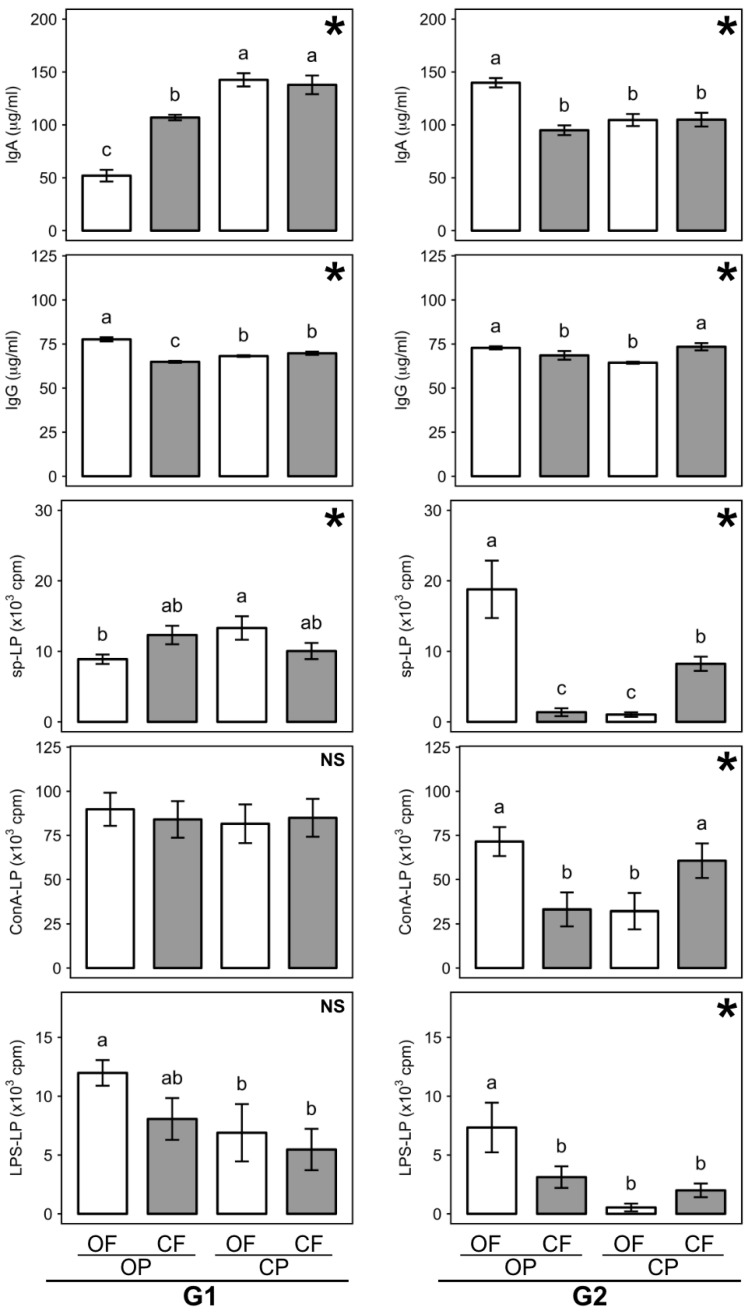
Effects of organic crop protection (OP) or conventional crop protection (CP) and organic fertility management (OF, white bars) or conventional fertility management (CF, shaded bars) on the plasma concentrations of immunoglobulin A (IgA) and immunoglobulin G (IgG), and on the lymphocyte proliferation: spontaneous (sp-LP), concanavalin A-stimulated (ConA-LP) and lipopolysaccharide-stimulated (LPS-LP) of the first (G1) and second (G2) generation of rats. Results shown as means ± SEM of *n* = 24 animals, different letters above bars indicate significant difference (*p* < 0.05) determined by Tukey’s HSD test. The interaction between agronomic factors indicated on the right upper corner of each plot as a star (*) when *p* < 0.05 or as NS when *p* ≥ 0.05. * Two-factor ANOVA detected a significant interaction between crop protection and fertilization regimes (see Table S6). Between crop protection and fertilization regimes used to produce feed crops for both IgA and IgG levels (Table S6; Figure 4), contrasting trends were found for interactions in the G1 and G2 (Figure 4).

Plasma immunoglobulin A (IgA) and G (IgG) levels in male rats were assessed nine weeks after weaning as markers for immune system capacity. We found no significant main effect of generation on IgA and IgG concentrations, but a range of significant interactions between rat generation, and crop protection and/or fertilization regimes used to produce feed crops (Figure 4; Table S4). Separate two-factor ANOVA tests for data from G1 and G2 rats were therefore carried out to further investigate these interactions (see below). Although two-factor ANOVA detected very highly significant interactions.

IgA levels in G1 rats consuming pesticide-treated feed crops (OFCP, CFCP) were significantly higher than those found in rats consuming untreated feed crops (OFOP, CFOP) (Table S6). Also, while the fertilization regime had no significant effect on IgA levels when pesticide-treated feed crops were compared, IgA levels were significantly lower in rats consuming manure-fertilized (OFOP) than mineral NPK-fertilized feed crops (CFOP) when untreated feed crops were compared in the G1 (Figure 4).

In contrast, in the G2, IgA levels in rats consuming pesticide-treated feed crops (OFCP, CFCP) were significantly lower than those found in rats consuming untreated feed crops (OFOP, CFOP) (Table S6). Also, while the fertilization regime had no significant effect on IgA levels when pesticide-treated feed crops were compared, IgA levels were significantly higher in rats consuming manure-fertilized (OFOP) than mineral NPK-fertilized feed crops (CFOP) when untreated feed crops were compared in the G2 (Figure 4). It is also important to point out, that in rats consuming organic (OFOP) feed, IgA levels were significantly lower in the G1, but higher in the G2, when compared to levels in rats consuming conventional feed (CFCP) (Figure 4).

IgG levels in G1 rats consuming pesticide-treated feed crops (OFCP, CFCP) were slightly, but significantly lower than those found in rats consuming untreated feed crops (OFOP, CFOP) (Table S6). Also, while the fertilization regime had no significant effect on IgG levels when pesticide-treated feed crops were compared, IgG levels were significantly higher in rats consuming manure-fertilized (OFOP) than mineral NPK-fertilised feed crops (CFOP) when untreated feed crops were compared in the G1 (Figure 4).

In contrast, in the G2, IgA levels in rats consuming pesticide-treated feed crops (OFCP, CFCP) were not significantly different compared to those found in rats consuming untreated feed crops (OFOP, CFOP) (Table S6). Also, when pesticide-treated feed crops were compared, IgG levels were significantly lower in rats consuming manure-fertilized (OFCP) than mineral NPK-fertilized (CFCP) feed crops, while IgA levels were significantly higher in rats consuming manure-fertilized (OFOP) than mineral NPK-fertilized (CFOP) feed crops when untreated feed crops were compared in the G2 (Figure 4).

It is also important to point out that in rats consuming organic (OFOP) feed, IgG levels were significantly higher in the G1, but similar in the G2, when compared to rats consuming conventional (CFCP) feed (Figure 4).

#### 3.6.2. Lymphocyte Proliferation

Lymphocyte proliferation (LP) tests were carried out using cell cultures established nine weeks after weaning. Three-factor ANOVA detected very highly significant main effects of generation on spontaneous proliferation (sp-LP) and proliferation levels in concanavalin A-stimulated (ConA-LP) and lipopolysaccharide-stimulated (LPS-LP) cell cultures (Table S4). In contrast, three-factor ANOVA detected no significant main effects of fertilization and a significant main effect (*p* = 0.03) of crop protection for LPS-LP only. Consumption of pesticide-treated feed crops (OFCP, CFCP) resulted in significantly lower LPS-LP in both the G1 and G2 (Figure 4; Table S6). However, significant three-way interactions between generation, and fertilization and crop protection regimes used for feed production for sp-LP and ConA-LP were also detected (Table S4). Separate two-factor ANOVA tests for data from G1 and G2 rats were therefore carried out to further investigate these interactions (see below).

Two-factor ANOVA detected significant interactions between crop protection and fertilization regimes for sp-LP in the G1 and G2 and for ConA-LP and LPS-LP in the G2 (Figure 4; Table S6). The interactions detected for sp-LP and ConA in the G2 were very highly significant (Table S5) and had a similar trend (Figure 4). However, for sp-LP the interactions detected in the G1 and G2 had contrasting trends (Figure 4).

In the G1, sp-LP was significantly higher when rats consumed pesticide-treated (OFCP) compared to untreated (OFOP) manure-fertilized feed crops, but significantly lower when rats consumed pesticide-treated (CFCP) compared to untreated (CFOP) mineral NPK-fertilized feed crops (Figure 4).

In contrast in the G2, sp-LP, ConA-LP and LPS-LP were significantly lower when rats consumed pesticide-treated (OFCP) compared to untreated (OFOP) manure-fertilized feed crops, but higher when rats consumed pesticide-treated (CFCP) compared to untreated (CFOP) mineral NPK-fertilized feed crops, although the difference between CFCP and CFOP was only significant for sp-LP and ConA-LP (Figure 4).

It is also important to point out that sp-LP (in the G2 only) and LPS-LP (in both the G1 and G2) were higher when rats consumed organic (OFOP) feed, when compared to levels in rats consuming conventional feed (CFCP) (Figure 4).

### 3.7. Associations Between Feed Composition, and Plasma Hormone and Immunological Parameters (RDA)

The relationships between the initial body weight and feed composition drivers, and plasma hormone profiles and immunological parameters were explored by redundancy analyses (RDA).

**Figure 5:**
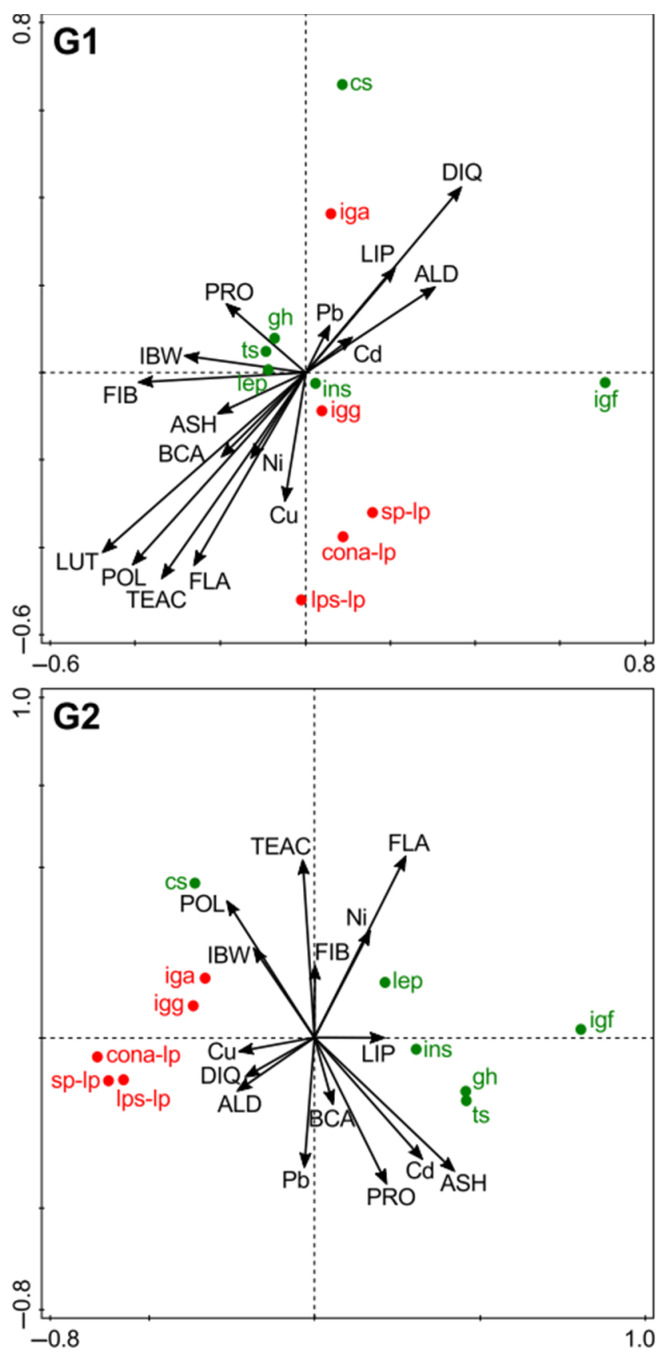
Biplot derived from the redundancy analysis (RDA) showing the relationships between concentrations of experimental feed components/parameters and selected hormones and immunological parameters in the first (G1) and second (G2) generation of rats. ASH, ash; LIP, lipids; PRO, protein; FIB, fiber; BCA, β-carotene; FLA, flavonols; LUT, lutein; POL, polyphenols; Cu, copper; ALD, aldicarb and DIQ, diquat; Cd, cadmium; Ni, nickel; Pb, lead; TEAC, feed antioxidant activity; IBW, initial body weight of rats at weaning, cs, corticosterone; gh, growth hormone; igf, insulin-like growth factor 1; lep, leptin; ins, insulin; ts, testosterone; iga, immunoglobulin A; igg, immunoglobulin G; sp-lp, spontaneous lymphocyte proliferation; cona-lp, concanavalin A-stimulated lymphocyte proliferation; lps-lp, lipopolysaccharide-stimulated lymphocyte proliferation.

For G1 rats, the horizontal axis 1 explained 38% of the variation, and the vertical axis 2 a further 10% (Figure 5). Feed concentrations of flavonols (F = 8.1, *p* = 0.002), lutein (F = 6.0, *p* = 0.007), ash (F = 2.9, *p* = 0.070) and protein (F = 2.6, *p* = 0.087) were identified as the strongest drivers. In comparison to these feed components, rat body weight at weaning (F = 1.9, *p* = 0.153) and antioxidant activity of feed (TEAC; F = 1.9, *p* = 0.153) explained less of the additional variation. Concentrations of fiber, lipids, polyphenols, β-carotene, copper (Cu), toxic metals (Cd, Ni, Pb) and residues of aldicarb and diquat also explained less (F ≤ 1.50, *p* ≥ 0.22) of the additional variance.

For G2 rats the horizontal axis 1 explained 58% of the variation, and the vertical axis 2 a further 2% (Figure 3). In the G2 polyphenols (F = 17, *p <* 0.001), ash (F = 5.6, *p* = 0.018), protein (F = 4.3, *p* = 0.033), nickel (F = 4.0, *p* = 0.038), flavonoids (F = 3.5, *p* = 0.063), and diquat residues (F = 3.2, *p* = 0.064) were identified as the strongest drivers. In comparison, body weight at weaning (F = 0.6, *p* = 0.52), and total antioxidant activity (TEAC), and concentrations of other nutrients (fiber, lipids, lutein), copper (Cu), toxic metals (Cd, Pb) and residues of aldicarb in feeds also explained less (F ≤ 1.9; *p* ≥ 0.15) of the additional variation.

#### 3.7.1. Plasma Hormone Profiles

For G1 rats there were positive associations between concentrations of IGF-1 (along the positive axis 1) and corticosterone (along the positive axis 2), and concentrations of diquat and aldicarb residues, and to a lesser extent lipids, Pb, and Cd in feeds. In contrast, no associations could be detected for the other hormones assessed (growth hormone, testosterone, insulin, and leptin) (Figure 5).

For G2 rats very different association patterns were identified by RDA. Plasma concentrations of IGF-1, insulin, growth hormone, testosterone, and leptin were all positively associated (along the positive axis 1) with concentrations of (i) protein, lipid, and ash, (ii) flavonoids, and (iii) cadmium and nickel in feeds. In contrast, plasma corticosterone concentrations were positively associated (along the negative axis 1) with concentrations of (i) polyphenols, (ii) copper, (iii) aldicarb, and diquat residues in feeds (Figure 5).

#### 3.7.2. Immunological Parameters

For G1 rats there were positive associations (along the positive axes 1 and 2) between IgA and concentrations of diquat and aldicarb residues, and to a lesser extent lipids in feeds. However, no associations could be detected for IgG (Figure 5).

For G2 rats both IgA and IgG showed the same association pattern. Similar to results for IgA in the G1, there were positive associations (along the negative axis 1) between plasma IgA and IgG and concentrations of diquat and aldicarb in feeds. However, different from the results for G1, in G2 there were also positive associations (along the negative axis 1) between plasma IgA and IgG concentrations and the initial weight at weaning, and concentrations of polyphenols and copper in feeds (Figure 5).

Associations between results from all three lymphocyte proliferation tests (spontaneous and ConA- or LPS-stimulated lymphocyte proliferation) and feed composition drivers were very similar (Figure 5).

For G1 rats, lymphocyte proliferation was positively associated (along the negative axis 2) with (i) antioxidant activity (TEAC) and concentrations of (ii) antioxidants (polyphenols, flavonoids, and lutein) and to a lesser extent (iii) β-carotene and (iv) Ni and Cu in feeds. For spontaneous and ConA-stimulated lymphocyte proliferation there were positive associations (along the positive axis 1) with concentrations of aldicarb and diquat residues, and to a lesser extent lipids, Cd, and Pb in feeds (Figure 5).

Similar, but not identical trends were also detected for G2 rats with positive associations (along the negative axis 1) between lymphocyte proliferation, and (i) initial body weight at weaning and (ii) antioxidant activity (TEAC), polyphenols aldicarb, and diquat residue levels in feeds (Figure 5).

### 3.8. Correlations between Endocrine and Immunological Parameters

Correlation analyses identified very different correlation patterns between the G1 and G2 and overall correlations were stronger in the G2 than the G1 (Figures S4–S9). Also, contrasting results (correlations being negative in one, but positive in the other generation) were detected between (i) Cs, and ConA-LP and LPS-LP, (ii) IGF-1, and IgA, IgG and sp-LP, insulin, (iii) insulin, and IgA and IgG, and (iv) leptin, and LPS-LP (Figures S4–S9).

In G2 rats, there were positive correlations between plasma Cs concentrations and all immunological parameters assessed, but negative associations between plasma concentrations of the other five hormones (GH, IGF-1, insulin, leptin, and Ts) and IgA, sp-LS, ConA-LS, and LPS-LS, except for a weak positive correlation between insulin and IgG (Figures S5 and S8).

## 4. Discussion

Although the confounding effects of lifestyle and diet composition can be controlled in animal model-based dietary intervention studies, there are major challenges for the design of dietary intervention studies related to the known effects of interactions between crop management parameters (e.g., fertilization and crop protection protocols used in organic and conventional cropping systems) on crop composition profiles [5,6,7,9,59,60,76,77]. For example, recent studies suggest that the use of pesticide-based crop protection regimes significantly reduce polyphenolic concentrations in crops fertilized with composted manure, but not in mineral NPK-fertilized crops [77]. Also, the use of pesticide-based crop protection regimes resulted in more than three times higher chlormequat residues in wheat crops fertilized with composted manure than in crops grown with mineral NPK fertilizers [59,77].

The unique factorial design of the dietary intervention study reported here allowed the effect of, and interactions between, different crop protection and fertilization regimes used in organic and conventional farming on crop composition and health-related physiological markers in rats to be identified. This study is also the first multigenerational study that compared the effect of feed composition differences resulting from organic and conventional crop protection and fertilization regimes on growth, hormone, and immune system parameters that were previously described as markers for the risk of overweight/obesity, metabolic syndrome and other chronic diseases in animals and humans [31,32,33,34,37,38,39,40,41,64,65,66,67].

Overall, the study confirms the results of a previous one-generation dietary intervention study with a similar design [59] which reported that composition differences resulting from the use of pesticides and mineral fertilizers in crop production have significant effects on plasma hormone profiles and immune system parameters in rats. In addition, the current study provides unique, indirect evidence for the hypothesis that differences in maternal nutrition (e.g., changes in protein, antioxidant, pesticide, and toxic metal intake) resulting from the use of agrochemicals result in epigenetic programming or the development of “adaptive” phenotypes in subsequent generations of rats.

By documenting interacting effects of mineral NPK fertilizer and pesticides (which are prohibited in organic farming) on crop composition and a range of health-related physiological parameters in two successive generations of rats, the study may also provide the foundation for elucidating the physiological mechanisms (e.g., changes in hormonal balances and immune systems responsiveness) underlying the associations between organic food consumption and lower incidences of obesity and other diseases reported in human cohort studies [46,47,48,49,50,51,52,53].

### 4.1. Effect of Crop Management Practices on Feed Composition

The composition differences between feeds made from organic (OFOP) and conventional (CFCP) crops found in this study were broadly consistent with results from a recent systematic literature review and meta-analysis [1], which reported higher antioxidant, but lower protein and cadmium concentrations, and a lower incidence of pesticide residues in organic crops. The differences in polyphenol and cadmium concentrations between the four feeds were also consistent with those reported previously in one generation factorial dietary intervention study with rats that also used feeds made from crops grown in the NFSC [59]. However, in this study, barley was the cereal component in feeds, while in the one-generation pilot study it was wheat. This explains why the plant growth regulator chlormequat (CCC; which is used to prevent lodging in conventional wheat, but not barley production) was not found as a pesticide residue in conventional feeds used in this study. Chlormequat, a confirmed endocrine-disrupting chemical, was only detected in pesticide-treated feed crops (OFCP and CFCP), but at significantly higher concentrations in manure-fertilized (OFCP) than NPK-fertilized (CFCP) wheat crops in the previous one-generation dietary intervention trial [59].

In contrast, the carbamate insecticide aldicarb and the herbicide diquat (which are both applied to the soil at planting and early in the growing season respectively) were detected in pesticide-treated feed crops (CFCP and OFCP) in the present study. Also, in this study pesticide residues were lower in crops fertilized with manure (OFCP) when compared to NPK-fertilised conventional (CFCP) crops. Due to the differences in pesticide residue profiles, it is difficult to compare and explain differences in outcomes between the pilot one-generation [59] and the two-generation rat study reported here.

In this study, the use of pesticides and mineral NPK for the production of feed crops resulted in higher feed intakes in both generations of rats. It is therefore important to note that not only differences in feed composition (resulting from contrasting agronomic regimes), but also feed intake by rats receiving contrasting feeds affected the total dietary intake for nutritionally-relevant compounds such as lipids, protein, fiber, pesticide, cadmium, lead, and antioxidants. Overall, the use of agrochemicals (pesticides and mineral NPK fertilizer) resulted in higher intakes of potentially harmful compounds and a lower intake of antioxidants that were previously linked to health benefits [78] in both generations of rats. Specifically, the use of pesticides for crop protection resulted in higher dietary intakes of pesticide residues and Cd, but lower intakes of polyphenols, flavonoids, and lutein, while the use of mineral NPK fertilizers resulted in higher protein and cadmium, but lower antioxidant intakes. When intakes from consumption of pesticide-treated crops were compared, NPK-fertilized feed crops (CFCP = conventional) also resulted in higher pesticide and Pb intakes than manure-fertilized feed crops (OFCP).

It should be pointed out that vitamin and mineral micronutrient supplements were added to all four rat feeds to comply with animal welfare regulations. The concentrations used greatly exceeded the relatively small differences in mineral micronutrient concentration detected between organic and conventional crops in previous studies (1,5,60). The use of supplements, therefore, resulted in standardized micronutrient concentrations in feeds, which was confirmed by the finding of similar Cu-concentrations in all four feeds.

However, since many of the micronutrients/vitamins also have antioxidant activity, the use of supplements may have also reduced the relative differences in antioxidant activity between feeds. This may explain why we detected substantial differences in concentrations of phenolic antioxidants, but no significant differences in total antioxidant activity (TEAC) between feeds.

### 4.2. Effects of Feed Composition Changes Resulting from Agrochemical Use on Basic Rat Physiological Parameters

White and red blood cell (WBC, RBC) count, packed cell volume (PCV), and plasma hemoglobin (Hb) levels were assessed as “toxicity” indicators, while blood glucose level and plasma antioxidant status (TEAC) were assessed as markers for energy metabolism and anti-oxidative stress capability respectively [79]. Although differences between rat generations and/or animals raised on the four contrasting feeds were detected, these were relatively small, which suggests that feed composition changes associated with agrochemical use did not cause acute stress, toxicity, and a major disruption of energy metabolism in rats. This was expected since pesticide (diquat and aldicarb) concentrations were below the maximum residue level (MRL) and toxic metal concentrations (Cd, Pb) below the maximum contamination level (MCL) set by the EU.

However, small but significant differences in RBC, Hb and glucose levels in G2 rats were found and these may have been due to (i) direct effects of differences in feed composition and intake and/or (ii) knock-on effects of changes in hormonal balances and immune system parameters in rats resulting from feed composition differences (see below). It is interesting to note that the only significant differences between rats consuming organic (OFOP) and conventional (CFCP) feeds were the slightly higher plasma TEAC (G1 rats only) and Hb levels (G2 rats only) found in rats consuming organic feed. The higher plasma antioxidant capacity (TEAC) levels may have been due to the higher polyphenol, flavonoid, and lutein intakes with organic feeds, and this view is supported by the positive correlations between plasma TEAC, and polyphenol, flavonoid, and lutein intake found in this study. Also, higher plasma Hb levels in rats consuming organic feed may be explained by higher iron (Fe) concentrations in organic crops, since plasma Hb levels are known to be affected by dietary Fe-intake [80]. This view is supported by the results of a recent retail survey which found significantly higher Fe concentrations in organic than conventional wholegrain wheat flour [60], although it should be pointed out that Fe concentrations in feeds were not assessed in this study, and barley was used as the cereal component in feeds.

#### 4.2.1. Plasma Hormone Concentrations

Plasma concentrations of corticosterone (Cs), growth hormone (GH), insulin-like growth factor 1 (IGF-1), insulin, leptin, and testosterone (Ts) were assessed in rats nine weeks after weaning. Hormone profiles were compared to identify (i) potential endocrine augmenting/disrupting effects of feed composition changes associated with pesticide and mineral fertilizer use and (ii) whether differences in maternal nutrition resulting from the consumption of contrasting feeds may result in epigenetic programming and/or “adaptive” phenotypes in subsequent generations.

ANOVA results showed that feed composition changes associated with pesticide and/or mineral fertilizer use (e.g., higher concentrations of aldicarb, diquat, and cadmium, but lower concentrations of fiber and antioxidants in feed crops) resulted in substantial differences in hormone profiles in rats. These findings are consistent with previous studies which reported endocrine and/or immune system disrupting effects of dietary exposure to pesticides and heavy metals such as Cd and Pb [21,32,33,34,37,40,41,67,81,82,83,84,85,86,87,88], and additive or synergistic effects of combinations of endocrine-disrupting pesticides (insecticides, herbicide and/or fungicides) [21,33,81,83,84].

Specifically, cadmium exposure was previously reported to stimulate steroidogenesis [37], which is consistent with the higher testosterone levels in G2 rats raised on mineral NPK instead of composted manure-fertilized feed crops produced without pesticides (which had the highest Cd levels, but no detectable aldicarb and diquat residues).

Similarly, the herbicides diquat and paraquat were reported to prevent the normal depletion of liver glycogen in starved rats and caused a significant increase in plasma corticosteroid concentrations within 24 h of exposure [81], which is consistent with the higher corticosteroid found in G1 rats raised on feeds made from pesticide-treated crops reported here.

Exposure to a combination of aldicarb, methomyl, and metribuzin pesticides (via the drinking water) was previously found to increase plasma GH concentrations in male rats after 13 weeks [41]. Also, wild rats, captured in banana plantations in which aldicarb and five organophosphorus pesticides (cadusafos, ethoprophos, isazophos, pyrimiphosethyl, terbufos) were applied, had lower testosterone concentrations and gonadosomatic indices (indicative of lower fertility) than rats captured in a National Park pesticide-free area [85]. However, in the study reported here, significant main effects of dietary pesticide exposure were only detected for corticosterone, IGF-1, and insulin, but not GH and Ts.

RDA results suggest that aldicarb, diquat, Cd, and Pb had similar (and possibly additive) effects on hormone concentrations and that these effects were modulated/partially reversed by antioxidant levels in feeds in both generations. These findings are consistent with previous studies which reported that (i) exposure to combinations of endocrine-disrupting chemicals (EDCs) (e.g., pesticides and Cd) can result in additive effects, (ii) that EDCs may affect the antioxidant status in animals, and (iii) increased dietary antioxidant/polyphenol/flavonoid intakes can affect both endocrine regulation (e.g., increased release of incretins, and, thus, insulin from pancreatic beta cells; improved insulin sensitivity) and immune system function [18,21,41,44,83,86,87,88].

Experimental studies with rodents suggest that differences in fiber, protein, and lipid intake may also result in changes in plasma hormone concentrations, including leptin, which is mainly secreted by adipocytes and has a vital role in body weight regulation by suppressing food intake and increasing energy expenditure [89]. For example, raising young male rats on high fiber or high protein diets resulted in a reduction in plasma leptin and insulin concentrations [90]. RDA results from this study also suggest that contrasting fiber, lipid, and especially protein intakes may have also contributed to changes in hormonal balances. However, different from previous studies with rats [90,91], leptin concentrations were positively associated with higher fiber and protein intake in both the G1 and G2. Also, it should be pointed out that the differences in fiber and/or protein concentrations and/or intakes between feeds used in the study reported here were very small compared to compared in previous studies [90,91].

The contrasting EDC, antioxidant, fiber, protein, and lipid concentration profiles generated by different feed crop management systems may, therefore, all have contributed to the large number of highly significant interactions between the two experimental factors (pesticide and mineral NPK fertilizer use in feed production) detected for individual hormone concentrations in both the G1 and G2.

The relative effect of feed composition differences (resulting from the use of pesticides and/or mineral NPK fertilizers) on plasma hormone profiles differed considerably between the first and second generations of rats. For example, RDA identified (i) pesticide residues in feeds as strong positive drivers for Cs and (ii) antioxidant activity and concentrations of polyphenols, flavonoids, and lutein in feeds as strong negative drivers for Cs, while in the G2 antioxidant activity and polyphenol concentrations in feed and initial body weight at weaning were identified as the strongest positive and Cd and protein content in feeds as the strongest negative drivers for plasma Cs concentrations. This may have been partially due to differences in maternal exposure and transfer of persistent toxic and/or endocrine-disrupting chemicals (e.g., Cd, Pb) to the next generation with milk during the suckling period [92,93]. For Cd, this view is supported by both the RDA (which identified Cd as a stronger driver for hormone concentrations in the G2 than the G1). However, there is also increasing evidence for transgenerational actions and epigenetic effects of EDCs [32,64]. Epigenetic programming and/or generation of “adaptive” phenotypes may therefore also have contributed to the differences in hormonal profiles between the G1 and G2.

It is important to highlight that no significant differences in hormone concentrations/profiles between rats raised on organic (produced without pesticides and mineral NPK fertilizer) and conventional (produced with both pesticides and mineral NPK fertilizer) feed crops were found in the G1 and that the only significant difference detected in G2 rats was a higher concentration of Cs in animals consuming organic feeds. Corticosterone is the main glucocorticoid (GC) involved in the regulation of energy, immune reaction, and stress responses in rodents. Glucocorticoids are known for their anti-inflammatory and immunosuppressive effects and are used in the treatment of inflammatory and autoimmune diseases, but long-term treatment with GCs was shown to increases the risk of osteoporosis, hypertension, insulin resistance, and type-2 diabetes [94].

It is also important to highlight that GH, IGF-1, insulin, leptin, and Ts concentrations in G2 rats raised on organic and conventional feeds were lower than those recorded in rats raised on feed crops treated with only one type of agrochemical (pesticides or mineral NPK). This may suggest that the increase in hormone concentrations resulting from feed composition changes associated with the use of mineral fertilizer might be partially neutralized/reversed by the feed composition changes associated with pesticide use and/or visa-versa.

#### 4.2.2. Immunological Parameters

Similar to the hormonal profiles, results from both the univariate analysis and RDA showed overall greater impacts and contrasting effects of feed composition differences in G2 than G1 rats for most immunological parameters. This provides further indirect evidence for epigenetic changes and/or the generation of “adaptive” phenotypes when consecutive generations of rats are raised on the same feed crops.

There is an increasing understanding of the integrated relationship and the mechanisms of bi-directional communication between the immune and neuro-endocrine systems and this has been reviewed extensively [94,95,96]. For example, changes in hormonal balances (including those associated with exposure to EDCs) are known to affect the immune system (e.g., lymphocyte proliferation) and immune cells such as lymphocytes can synthesize and secrete several hormones (e.g., GH) with immunomodulatory properties [94,96]. This close integration is thought to allow the brain to modulate immune system activity, and thereby keep the homeostasis of the whole body in an appropriate manner [94,95,96]. The finding of feed composition differences (e.g., EDC, antioxidant, and protein/lipid/protein concentrations) that affected the endocrine system also had a substantial impact on immune system parameters was therefore not surprising.

Results from this study also indicated a close interrelationship between the endocrine and immune system responses to the four contrasting feed composition profiles. For example, for both endocrine and immune system parameters assessed, interactions between mineral NPK fertilizer and pesticide use were detected for only some parameters in the G1, but all endocrine and immune parameters in the G2. Also, the trends of the interactions for plasma IgG, sp-LP, and ConA-LP in G2 rats represent a mirror image of the interactions found for all plasma hormones except Cs in the G2. Specifically, lower IgG levels, sp-LP, and LPS-LP were found in rats consuming feed crops produced with only one type of agrochemical (mineral NPK fertilizer or pesticides; OFCP, CFOP) compared to rats consuming conventional (CFCP) or organic (OFOP) feed crops. As with many hormones in the G2, this suggests, that lower plasma IgG concentrations and lymphocyte proliferation resulting from feed composition changes associated with the use of mineral fertilizer were at least partially neutralized/canceled out by the composition changes associated with pesticide use and/or visa-versa.

This study also found that feed composition differences had larger and for some endocrine and immunological parameters contrasting effects in the G2 compared to the G1. This could have been due to endocrine and immunological parameters being driven primarily by feed composition in the G1, while in the G2 both (i) feed composition differences and (ii) epigenetic changes and/or the development of “adaptive” phenotypes (induced by maternal diets during early fetal development) contributed to the overall effects observed.

Results from univariate and correlation analyses suggest that the differences in immune parameters between the G1 and G2 were primarily driven by endocrine regulatory mechanisms involving Cs, IGF-1, and insulin. Specifically, this view is supported by the findings that (i) in the G1 only Cs, IGF-1, and insulin concentrations were affected by feed composition, while in the G2 significant differences were detected for all six hormones assessed, (ii) feed composition had contrasting effects on plasma Cs, IGF-1 and to a lesser extent insulin concentrations in the G1 and G2, and (iii) contrasting correlations between endocrine and immune parameters in G1 and G2 rats were only detected for Cs, IGF-1 and insulin.

In G2 rats, there were positive correlations between plasma Cs concentrations and negative associations between plasma concentrations of the other five hormones (GH, IGF-1, insulin, leptin, and Ts), and IgA, sp-LP, ConA-LP, and LPS-LP. This and the finding that in the G2 the only significant differences between rats raised on organic and conventional feeds were higher Cs and IgA, sp-LP, and LPS-LP in rats consuming organic feed suggest that Cs might have had a particularly important role in the regulation of immune parameters. However, while the negative correlation between plasma Cs and stimulated LP (ConA and LPS) found in G1 rats are consistent with the immunosuppressive effects of corticosteroids reported in previous animal and human studies [94], the strong positive correlations between Cs and LP identified in the G2 differ from those reported in previous studies [94].

The differences in both hormonal balances and immune system parameters in rats raised on contrasting feeds detected in this study may therefore have been due to a combination of (i) direct effects of feed crop composition parameters (e.g., endocrine disrupting effect and immunosuppressing effects of Cd and pesticides exposure or protective effects of dietary antioxidants or changes in plasma leptin and insulin concentrations triggered by higher fiber or lipid intake), (ii) indirect effects resulting from the communication between the immune and neuroendocrine systems in response to differences in feed crop composition [95,96] and/or (iii) epigenetic changes and the development of “adaptive” phenotypes in the G2.

#### 4.2.3. Rat Growth Parameters

Similar to the pattern found for physiological parameters (see above), composition differences resulting from contrasting fertilization and crop protection regimes used for feed crop production resulted in contrasting effects on growth parameters in G1 and G2 rats. Specifically, trends toward main effects of fertilization regimes were only detected in the G1 (higher feed intake and total weight gain in rats consuming mineral NPK-fertilized feed crops), while significant main effects of crop protection (higher initial body weight at weaning, feed intake and feed conversion ratio in rats consuming pesticide-treated feed crops) were only detected in the G2.

Previous studies in model animals and/or humans suggest that higher body weights at weaning, feed/food intake, and weight gain early in life can be markers/risk factors for obesity and metabolic syndrome later in life, while higher antioxidant intakes were shown to be linked to lower risks of obesity and associated diseases including cardiovascular diseases and certain cancers [63,97,98,99,100,101,102,103,104].

The findings of (i) higher feed intake and body weights in G1 consuming mineral NPK fertilized feed crops, (ii), higher initial body weights at weaning and feed intake in G2 rats consuming pesticide-treated feed crops, and (iii) lower antioxidant intakes resulting from both types of agrochemical use may therefore all indicate a higher risk of overweight/obesity and associated diseases at later stages of development. This view is supported by the finding that consumption of conventional feeds (which are produced with both types of agrochemicals) results in nutritional changes (higher dietary intakes of protein, lipid, and Cd and reduced intakes of polyphenols) which have all been reported as risk factors for overweight/obesity, metabolic syndrome and associated diseases [24,63,65,66,105].

### 4.3. Evidence for Epigenetic Changes and/or Development of ‘Adaptive’ Phenotypes

Epigenetic regulation is now recognized as a fundamental property of eucaryotic genomes [106]. Epigenetic programming influences the expression of genes without altering the DNA sequence and leads to heritable changes in phenotypic expression [107,108]. The known molecular mechanisms of epigenetic modifications include DNA methylation [109], histone modifications [110], aberrant microRNA (miRNA) expression [111], and even protein-protein interactions [112]. These mechanisms have been confirmed to have a strong impact on the development, health, and reproduction of humans [108].

The epigenome was shown to be altered by hormones, environmental factors, age, but also by a number of nutritional factors including dietary exposure to EDCs, and the impacts of such alterations can be observed across generations [108]. Although perinatal time is the period of highest phenotypic plasticity, largely impacting developmental programming, there is also evidence that dietary factors influence epigenetic regulation in adults [113].

Maternal undernutrition and overnutrition can affect the epigenome of the fetus, and these effects can be observed throughout the lifespan [108]. Diseases known to be controlled by the hormonal system, such as diabetes and obesity, as well as female infertility, have also been reported to be associated with epigenetic changes [108]. These phenotypes can be seen not only in F1 but can be passed through the germline, having transgenerational effects, expressed in the “adaptive” phenotypes of F3 [108].

It is important to point out that in the study presented here feed intake, and weight gain were only assessed for nine weeks after weaning and in only two successive generations of rats, while studies focused on assessing transgenerational effects of nutritional factors on obesity and other diseases usually monitor rats until later in life and for three or more generations [24,62,63,64]. However, the effects of the four different feed crops on rat growth endocrine and immunological parameters that are known to affect (and/or are affected by) weight gain, obesity, and/or associated disease were very different in the G1 and G2. This finding is consistent with those described in recent multigenerational studies in rodents that reported transgenerational epigenetic transmission of phenotypes resulting from maternal diet. For example, the effects of high-fat diets on body weight and glucose tolerance were shown to be inherited and/or accelerated in subsequent generations [62,63].

There is also increasing evidence that other maternal dietary factors (including several nutrient deficiencies, intakes of EDCs including pesticides, omega-3 fatty acids, poly-phenols, and other phytochemicals) may result in epigenetic programming of immune and endocrine functions and affect the risk of a number of diseases, including atopic disease and cancer development in subsequent generations [114,115,116]. Nutritional factors have been shown to impact DNA methylation reactions [117,118] and nutrient deficiency can lead to induction of DNA hypomethylation with consequences such as i.e., cancer development [119].

In the context of the results of the study reported here, it is important to consider that polyphenols such as catechins have been shown to influence the epigenome through several mechanisms [120]. For example, in vitro studies reported that green tea catechins were shown to decrease the DNA methylation level of gene promoters involved in DNA reparation, thus restoring their functions [121]. Also, consumption of black raspberry fruit with a high phenolic content was observed to result in the DNA demethylation of gene promoters involved in colorectal cancer proliferation [122]. Epigenetic regulatory mechanisms may therefore at least partially explain cancer-preventing effects linked to the consumption of (poly)phenolic-rich foods/feed crops [123,124].

### 4.4. Study Limitations

The main limitations of this study were (i) the short observation period post weaning, (ii) that only two successive generations of rats were assessed, and (iii) the lack of assessments of epigenetic changes.

Other limitations were that we only assessed Cu-concentrations in feeds and estimated pesticide exposure from crop rather than urine analysis, although there is evidence that micro-nutrients are higher in organic than conventional crops [60], and a recent human dietary intervention study demonstrated that urine analyses provide a more accurate estimate of total pesticide exposure and pesticide profiles than crop analyses based on multi-residue test methods (Rempelos et al., submitted for publication). Also, we added mineral micronutrient supplements to all four rat feeds at concentrations that greatly exceeded the small differences in mineral micronutrient concentration found between organic and conventional crops (60), This made it was impossible to study the effects of differences in mineral micronutrient concentrations between feeds crops on rat physiology.

As a result, some potentially important nutritional drivers (e.g., Fe, Zn, Se) could not be included in the RDA and it is likely that differences in pesticide residues levels between feeds were underestimated.

It should be pointed out that the insecticide aldicarb is now prohibited in the EU, but is still used in the US and a range of other countries outside the EU [125].

## 5. Conclusions

Results of this factorial, two-generation rat dietary intervention study suggest that the combination of the relatively small changes in dietary intakes of (i) protein, lipids, and fiber, (b) toxic and/or endocrine-disrupting pesticides and metals, (iii) polyphenols and other antioxidants, resulting from pesticide and/or mineral NPK fertilizer use in feed crop production, can have complex, interactive effects on endocrine, immunological and growth parameters that are known to affect the risk of overweight/obesity, metabolic syndrome and other chronic diseases in animals and humans. However, in the study reported here, growth and other physiological parameters were only monitored for 9 weeks after weaning. It was therefore not possible to determine whether and to what extent (i) the differences in feed composition, (ii) dietary intakes of compounds previously linked to obesity and chronic diseases, and/or (iii) the changes in endocrine and immune parameters in rats raised on feed crops treated with mineral fertilizers and/or pesticides, would have resulted in higher levels of weight gain and/or diseases linked to obesity, endocrine disruption and/or changes in immune system activity/responsiveness. However, results provide, for the first time, indirect evidence for transgenerational, epigenetic changes and/or the development of “adaptive” phenotypes in the second generation of rats.

Before drawing conclusions on potential health impacts of composition changes resulting from organic and conventional farming practices, it is therefore important to carry out additional multi-generation studies with rodent models to confirm results and to study growth gain, endocrine, and immunological parameters throughout the lifespan of animals. Also, in order to identify and understand the effects of feed composition changes on obesity and other chronic diseases, both standard (e.g., the Wistar Cmd: WI [WU] used here) and obesity/disease model rodent strains [126,127,128] should be included in studies and the range of assessments should be expanded to other health-related physiological markers (e.g., oxidative stress and inflammation-related markers).

Nonetheless, if results are confirmed and differences in the risk of obesity and other health outcomes can be demonstrated, the factorial experimental approach in this study could provide the basis for a mechanistic understanding of the lower risk of obesity, metabolic syndrome, cancer, and other diseases associated with organic food consumption in recent human dietary intervention studies [46,47,48,49,50,51,52,53,54,55].

## Figures and Tables

**Table 1:** Effect of crop protection and fertilization on the growth parameters of the first (G1) and second (G2) generation rats. Values shown are means ± SE.

Factor	Initial Body Weight at Weaning (g)		Feed Intake (g/day)		Total Weight Gain (g)		Feed Conversion Ratio (%)	
	G1	G2	G1	G2	G1	G2	G1	G2
Crop protection								
Organic (-pesticides)	60 ± 1	95 ± 5	18.3 ± 0.3	16.9 ± 0.3	263 ± 3	221 ± 4	4.73 ± 0.03	4.75 ± 0.11
Conventional (+pesticides)	62 ± 1	114 ± 2	18.6 ± 0.2	17.8 ± 0.2	266 ± 3	219 ± 3	4.74 ± 0.03	4.99 ± 0.05
Fertilization								
Organic (manure)	61 ± 1	110 ± 4	17.9 ± 0.2	17.3 ± 0.2	256 ± 3	209 ± 3	4.73 ± 0.03	5.10 ± 0.09
Conventional (mineral NPK)	62 ± 1	99 ± 4	19.0 ± 0.2	17.4 ± 0.3	273 ± 3	230 ± 4	4.74 ± 0.03	4.65 ± 0.08
ANOVA results								
(*p*-values *)								
Main effects								
Crop protection (P)	0.200	**<0.001**	0.158	**0.002**	0.360	0.924	0.703	**0.020**
Fertilisation (F)	0.644	0.507	*0.050*	0.856	*0.050*	0.141	0.985	0.184
Interaction (PxF)	0.984	0.256	0.080	0.485	0.680	0.356	0.006	0.664

* *p*-values in bold are for significant differences (*p* < 0.05); *p*-values in *italic* are for trends (0.1 > *p* > 0.05); NPK, nitrogen, phosphorus and potassium.

## Data Availability

Data will be made available upon reasonable request by author Marcin Baranski.

## Supplementary Materials

The following are available online at https://www.mdpi.com/2072-6643/13/2/377/s1, Table S1. Previous crops, planting and harvesting dates, crop protection and fertilization regimes used for the barley, potato, carrot and onion feed crop cultivation; Table S2. Effect of crop protection and fertilization on the chemical composition of the experimental rat feeds (per 100g fresh weight); Table S3. Effect of crop protection and fertilization on the daily intake of macronutrients, antioxidants, contaminants and minerals in the first and second generation of rats; Table S3 continued. Effect of crop protection and fertilization on the daily intake of macronutrients, antioxidants, contaminants and minerals inthe first and second generation of rats; Table S4. Results of the 3-factor ANOVA of effects of crop protection(P), fertilization(F)and generation (G)on (a)growth, basic physiological, endocrine and immunological parameters and (b)dietary macronutrient, antioxidant and contaminant intake in rats; Table S4 continued. Results (*p*-values) of the 3-factor ANOVA of effects of crop protection, fertilization and generation on (a)growth, basic physiological, endocrine and immunological parametersand (b)dietary macronutrient, antioxidant andcontaminant intake in rats; Table S5. Rat body composition and basic physiological parameters in the first and second generation of rats; Table S6. Effect of crop protection and fertilization on plasma hormones, immunoglobulin concentrations and immune system responsiveness in the first and second generation of rats Figure S1 Experimental design flow diagram. * animals assessed in trials; Figure S2. Effects of organic crop protection (OP) or conventional crop protection (CP) and organic fertility management (OF) or conventional fertility management (CF) on the daily intake of protein, lipid, fiber, ash, Cu, polyphenols, flavonoids, lutein, β-carotene, antioxidant capacity (TEAC) of the first (G1) and second (G2) generation of rats. Results shown as means ±SEM of *n* = 24 animals, different letters above bars indicate significant difference (*p* ≤ 0.05) determined by Tukey’s HSD test. * two factor ANOVA detected a significant interaction between crop protection and fertilization regimes (see Table S3 for ANOVA *p*-values and main effect means); Figure S3. Effects of organic crop protection (OP) or conventional crop protection (CP) and organic fertility management (OF) or conventional fertility management (CF) on the daily intake of cadmium (Cd) and lead (Pb) of the first (G1) and second (G2) generation of rats. Results shown as means ±SEM of *n* = 24 animals, different letters above bars indicate significant difference (*p* ≤ 0.05) determined by Tukey’sHSD test. * two factor ANOVA detected a significant interaction between crop protection and fertilization regimes (see Table S3 for ANOVA *p*-values and main effect means); Figure S4. Correlation between concentration of experimental feed components and physiological parametersfor the first generation of rats (G1). The Pearson correlation was indicated on the color scale(blue as positive and red as negative), where color intensity and circle sizes are proportional to the correlation coefficient. PRO, protein; LIP, lipids; FIB, fiber; ASH, ash; POL, polyphenols; FLA, flavonols; LUT, lutein; BCA, β-carotene; TEAC, feed antioxidant activity; Cu, copper; ALD, aldicarb;DIQ, diquat; Cd, cadmium; Ni, nickel; Pb, lead; CONTAM, contaminants altogether; IBW.G1, initial body weightof first generation of rats; IBW.G2, initial body weight of second generation of rats;int, total feed intake; twg, total weight gain; FCR, feed conversion ratio; %BDM, percent of body dry matter; rbc, red blood cells count; hb, blood hemoglobin content; glu, plasma glucose content; teac, plasma antioxidant capacity; cs, corticosterone; gh, growth hormone; igf, insulin-like growth factor 1; ins, insulin; lep, leptin; ts, testosterone; iga, immunoglobulin A; igg, immunoglobulin G; sp-lp, spontaneous lymphocyte proliferation; cona-lp, concanavalin A-stimulated lymphocyte proliferation; lps-lp, lipopolysaccharide-stimulated lymphocyte proliferation; Figure S5. Correlation between concentration of experimental feed components and physiological parameters for the second generation of rats (G2). The Pearson correlation was indicated on the color scale (blue as positive and red as negative), where color intensity and circle sizes are proportional to the correlation coefficient. PRO, protein; LIP, lipids; FIB, fiber; ASH, ash; POL, polyphenols; FLA, flavonols; LUT, lutein; BCA, β-carotene; TEAC, feed antioxidant activity; Cu, copper; ALD, aldicarb; DIQ, diquat; Cd, cadmium; Ni, nickel; Pb, lead; CONTAM, contaminants altogether; IBW.G2, initial body weight of second generationof rats; int, total feed intake; twg, total weight gain; FCR, feed conversion ratio; %BDM, percent of body dry matter; rbc, red blood cells count; hb, blood hemoglobin content; glu, plasma glucose content; teac, plasma antioxidant capacity; cs, corticosterone; gh, growth hormone; igf, insulin-like growth factor 1; ins, insulin; lep, leptin; ts, testosterone; iga, immunoglobulin A; igg, immunoglobulin G; sp-lp, spontaneous lymphocyte proliferation; cona-lp, concanavalin A-stimulated lymphocyte proliferation; lps-lp, lipopolysaccharide-stimulated lymphocyte proliferation; Figure S6. Correlation between concentration of experimental feed components and physiological parameters for both generations of rats (G1+G2). The Pearson correlation was indicated on the color scale (blue as positive and red as negative),where color intensity and circle sizes are proportional to the correlation coefficient. PRO, protein; LIP, lipids; FIB, fiber; ASH, ash; POL, polyphenols; FLA, flavonols; LUT, lutein; BCA, β-carotene; TEAC, feed antioxidant activity; Cu, copper; ALD, aldicarb; DIQ, diquat; Cd, cadmium; Ni, nickel; Pb, lead; CONTAM, contaminants altogether; IBW.G1, initial body weight of first generation of rats; IBW, initial body weight of rats; int, total feed intake; twg, total weight gain; FCR, feed conversion ratio; %BDM, percent of body dry matter; rbc, red blood cells count; hb, blood hemoglobin content; glu, plasma glucose content; teac, plasma antioxidant capacity; cs, corticosterone; gh, growth hormone; igf, insulin-like growth factor 1; ins, insulin; lep, leptin; ts, testosterone; iga, immunoglobulin A; igg, immunoglobulin G; sp-lp, spontaneous lymphocyte proliferation; cona-lp, concanavalin A-stimulated lymphocyte proliferation; lps-lp, lipopolysaccharide-stimulated lymphocyte proliferation; Figure S7. Correlation between calculated intake of experimental feed components and physiological parameters for the first generation of rats (G1). The Pearson correlation was indicated on the color scale (blue as positive and red as negative), where color intensity and circle sizes are proportional to the correlation coefficient. PRO, protein; LIP, lipids; FIB, fiber; ASH, ash; POL, polyphenols; FLA, flavonols; LUT, lutein; BCA, β-carotene; TEAC, feed antioxidant activity; Cu, copper; ALD, aldicarb; DIQ, diquat; Cd, cadmium; Ni, nickel; Pb, lead; CONTAM, contaminants altogether; IBW.G1, initial body weight of first generation of rats; IBW.G2, initial body weight of second generation of rats; int, total feed intake; twg, total weight gain; FCR, feed conversion ratio; %BDM, percent of body dry matter; rbc, red blood cells count; hb, blood hemoglobin content; glu, plasma glucose content; teac, plasma antioxidant capacity; cs, corticosterone; gh, growth hormone; igf, insulin-like growth factor 1; ins, insulin; lep, leptin; ts, testosterone; iga, immunoglobulin A; igg, immunoglobulin G; sp-lp, spontaneous lymphocyte proliferation; cona-lp, concanavalin A-stimulated lymphocyte proliferation; lps-lp, lipopolysaccharide-stimulated lymphocyte proliferation; Figure S8. Correlation between calculated intake of experimental feed components and physiological parameters for the second generation of rats (G2). The Pearson correlation was indicated on the color scale (blue as positive and red as negative), where color intensity and circle sizes are proportional to the correlation coefficient. PRO, protein; LIP, lipids; FIB, fiber; ASH, ash; POL, polyphenols; FLA, flavonols; LUT, lutein; BCA, β-carotene; TEAC, feed antioxidant activity; Cu, copper; ALD, aldicarb; DIQ, diquat; Cd, cadmium; Ni, nickel; Pb, lead; CONTAM, contaminants altogether; IBW.G2, initial body weight of second generationof rats; int, total feed intake; twg, total weight gain; FCR, feed conversion ratio; %BDM, percent of body dry matter; rbc, red blood cells count; hb, blood hemoglobin content; glu, plasma glucose content; teac, plasma antioxidant capacity; cs, corticosterone; gh, growth hormone; igf, insulin-like growth factor 1; ins, insulin; lep, leptin; ts, testosterone; iga, immunoglobulin A; igg, immunoglobulin G; sp-lp, spontaneous lymphocyte proliferation; cona-lp, concanavalin A-stimulated lymphocyte proliferation; lps-lp, lipopolysaccharide-stimulated lymphocyte proliferation; Figure S9. Correlation between calculated intake of experimental feed components and physiological parameters for both generations of rats (G1+G2). The Pearson correlation was indicatedon the color scale (blue as positive and red as negative), where color intensity and circle sizes are proportional to the correlation coefficient. PRO, protein; LIP, lipids; FIB, fiber; ASH, ash; POL, polyphenols; FLA, flavonols; LUT, lutein; BCA, β-carotene; TEAC, feed antioxidant activity; Cu, copper; ALD, aldicarb; DIQ, diquat; Cd, cadmium; Ni, nickel; Pb, lead; CONTAM, contaminants altogether; IBW.G1, initial body weight of first generation of rats; IBW, initial body weight of rats; int, total feed intake; twg, total weight gain; FCR, feed conversion ratio; %BDM, percent of body dry matter; rbc, red blood cells count; hb, blood hemoglobin content; glu, plasma glucose content; teac, plasma antioxidant capacity; cs, corticosterone; gh, growth hormone; igf, insulin-like growth factor 1; ins, insulin; lep, leptin; ts, testosterone; iga, immunoglobulin A; igg, immunoglobulin G; sp-lp, spontaneous lymphocyte proliferation; cona-lp, concanavalin A-stimulated lymphocyte proliferation; lps-lp, lipopolysaccharide-stimulated lymphocyte proliferation.
