# Supplementary material for: Feed Composition Differences Resulting from Organic and Conventional Farming Practices Affect Physiological Parameters in Wistar Rats—Results from a Factorial, Two-Generation Dietary Intervention Trial
Source: Nutrients. 2021 Jan 26;13(2):377. doi: 10.3390/nu13020377 (PMC7911726; doi:10.3390/nu13020377)
Supplement: Supplementary file 1 [file nutrients-13-00377-s001.pdf]

Supplementary material

Table S1. Previous crops, planting and harvesting dates, crop protection and fertilization regimes used for the barley, potato, carrot and onion feed crop cultivation

	Barley	potato	carrot	onion
Variety	Pearl	Sante	Nairobi	Hyfort
Previous crops	winter wheat	winter barley	winter barley	winter barley
**Fertility Management (F)**				
Organic (OF)	no input	composted cattle manure to equivalent to 170 kg N/ha	composted cattle manure to equivalent to 85 kg N/ha	composted cattle manure to equivalent to 170 kg N/ha
Conventional (CF)	ammonium-nitrate (Nitram): 50 kg N/ha in mid March and 120 kg N/ha mid April to equivalent to 170 kg N/ha, and Superphosphate and KCL as compound fertilizer (0:20:30) to equivalent to 64 kg P/ha and 96 kg K/ha	ammonium-nitrate (Nitram) to equivalent to 180 kg N/ha, and Superphosphate and KCL as compound fertilizer (0:20:30) to equivalent to 134 kg P/ha and 200 kg K/ha	ammonium-nitrate (Nitram) to equivalent to 20 kg N/ha, and Superphosphate and KCL as compound fertilizer (0:20:30) to equivalent to 100 kg P/ha and 150 kg K/ha	ammonium-nitrate (Nitram) to equivalent to 75 kg N/ha, and Superphosphate and KCL as compound fertilizer (0:20:30) to equivalent to 100 kg P/ha and 150 kg K/ha
**Crop Protection (P)**				
Organic (OP)				
weed control	2 × mechanical weeding	4 × ridging	2-3 × hand-hoeing	5-6 × mechanical weeding
pest control	-	-	netting	-
disease control	-	copper oxychloride; 4 application per year	-	-
Conventional (CP)				
weed control	tank mix (applied in November): pendimethalin (2.5 L/ha), isoproturon (3 L/ha), mecoprop-P (1 L/ha); fluroxypyr (0.75 L/ha) applied in April	linuron (3.5 L/ha, 1 application per year), diquat (2 applications per year)	linuron (3.5 L/ha, 1 application per year)	propachlor (9 L/ha, 1 application per year)
pest control	-	aldicarb (33.6 kg/ha, 1 application per year)	aldicarb (33.6 kg/ha, 1 application per year)	-
disease control	1st tank mix (applied in April): cyprodinil and picoxystrobin (0.4 L/ha), prothioconazole (0.4 L/ha), fenpropimorph (0.25 L/ha); 2nd tank mix (applied in May):	fluazinam (3 applications per year), metalaxyl-M and mancozeb (3 applications per year)	azoxystrobin (1 L/ha, 3 applications per year), epoxiconazole (1 L/ha, 2 applications per year)	azoxystrobin (1 L/ha, 3 applications per year), iprodione (2 L/ha, 3 applications per year)

azoxystrobin (0.25 L/ha),
chlorothalonil (0.5 L/ha),
epoxiconazole (0.4 L/ha),
fenpropimorph (0.125 L/ha)

Table S2. Effect of crop protection and fertilization on the chemical composition of the experimental rat feeds (per 100 g fresh weight)

Feed components	Crop protection (P)				Fertility management (F)				ANOVA results (*P* values*)		
	Organic (- pesticides)		Conventional (+ pesticides)		Organic (composted manure)		Conventional (mineral NPK fertilizer)				
	Mean	SEM	Mean	SEM	Mean	SEM	Mean	SEM	P	F	P×F
**Macronutrients**											
Protein (g)	21.2	0.4	20.7	0.4	20.2	0.4	21.8	0.3	0.342	*0.053*	0.574
Lipid (g)	5.64	0.29	6.16	0.17	5.68	0.26	6.12	0.21	**0.026**	*0.088*	0.144
Fiber (g)	2.81	0.06	2.59	0.02	2.73	0.07	2.67	0.05	**0.014**	0.436	0.970
Ash (g)	5.75	0.07	5.73	0.03	5.68	0.06	5.80	0.05	0.755	0.294	0.144
**Antioxidants**											
Polyphenols (mg)	1682	104	1199	73	1663	112	1218	77	**<0.001**	**0.004**	**0.002**
Flavonoids (mg)	2.86	0.18	2.39	0.14	2.87	0.10	2.37	0.19	**0.028**	0.105	0.514
Lutein (mg)	0.36	0.04	0.25	0.03	0.33	0.03	0.28	0.04	**0.016**	0.171	0.405
β-carotene (mg)	0.73	0.05	0.69	0.07	0.74	0.07	0.68	0.05	0.569	0.664	0.631
TEAC (μmol TE)	25.5	0.2	25.1	0.2	25.5	0.2	25.1	0.2	0.106	0.208	0.690
**Minerals**											
Nitrogen (N; g)	3.40	0.07	3.32	0.07	3.23	0.06	3.48	0.05	0.334	*0.053*	0.585
Copper (Cu; μg)	0.256	0.006	0.250	0.007	0.256	0.006	0.250	0.007	0.180	0.647	*0.069*
**Contaminants**											
Aldicarb (μg)	0.00	0.00	3.11	1.11	0.76	0.37	2.34	1.27	**0.018**	0.199	0.152
Diquat (μg)	0.00	0.00	0.27	0.10	0.08	0.06	0.18	0.10	**0.032**	0.390	0.355
Cadmium (Cd; μg)	3.20	0.31	3.20	0.39	2.35	0.12	4.05	0.16	0.996	**0.004**	0.556
Nickel (Ni; μg)	7.52	0.82	6.85	0.58	7.50	0.77	6.86	0.65	0.529	0.565	0.957
Lead (Pb; μg)	3.69	0.58	4.27	0.76	3.51	0.50	4.46	0.79	0.561	0.385	0.284

TEAC, trolox equivalent antioxidant capacity; TE, trolox equivalent. *P-values in **bold** are for significant differences (P<0.05); P-values in *italic* are for trends (0.1>P>0.05)

Table S3. Effect of crop protection and fertilization on the daily intake of macronutrients, antioxidants, contaminants and minerals in the first and second generation of rats

		Crop protection (P)				Fertility management (F)						
		Organic (- pesticides)		Conventional (+ pesticides)		Organic (composted manure)		Conventional (mineral NPK fertilizer)		ANOVA results (*P* values*)		
**Feed components**	G	Mean	SEM	Mean	SEM	Mean	SEM	Mean	SEM	P	F	P×F
**Macronutrients**												
Protein (g)	1	3.88	0.07	3.87	0.05	3.61	0.04	4.13	0.05	0.874	**0.006**	0.420
	2	3.60	0.05	3.70	0.05	3.50	0.04	3.79	0.05	0.110	**0.018**	0.116
Lipid (g)	1	1.03	0.03	1.14	0.02	1.01	0.02	1.16	0.02	**<0.001**	**0.004**	**<0.001**
	2	0.95	0.02	1.10	0.02	0.98	0.03	1.06	0.01	**<0.001**	*0.096*	0.125
Fiber (g)	1	0.51	0.01	0.48	0.00	0.49	0.01	0.51	0.01	**<0.001**	0.105	*0.098*
	2	0.48	0.01	0.46	0.00	0.47	0.01	0.46	0.01	*0.067*	0.615	0.407
Ash (g)	1	1.05	0.02	1.07	0.01	1.01	0.01	1.10	0.01	0.294	**0.038**	**0.008**
	2	0.98	0.01	1.02	0.01	0.99	0.01	1.01	0.01	**0.003**	0.420	0.872
**Antioxidants**												
Polyphenols (mg)	1	306	7	222	5	299	7	231	6	**<0.001**	**0.012**	*0.069*
	2	282	8	215	5	286	8	213	5	**<0.001**	**0.003**	**<0.001**
Flavonoids (mg)	1	0.53	0.02	0.44	0.01	0.51	0.01	0.46	0.02	**<0.001**	0.288	**0.002**
	2	0.49	0.02	0.43	0.01	0.50	0.01	0.42	0.02	**<0.001**	0.203	0.152
Lutein (mg)	1	0.07	0.00	0.05	0.00	0.06	0.00	0.05	0.00	**<0.001**	0.307	**<0.001**
	2	0.061	0.003	0.045	0.002	0.057	0.002	0.049	0.003	**<0.001**	0.315	**0.008**
β-carotene (mg)	1	0.13	0.00	0.13	0.01	0.13	0.01	0.13	0.00	0.260	0.985	**0.037**
	2	0.12	0.00	0.12	0.01	0.13	0.01	0.12	0.00	0.889	0.690	0.129
TEAC	1	4.66	0.07	4.66	0.05	4.55	0.05	4.77	0.06	0.855	0.181	0.131
(μmol TE)	2	4.32	0.07	4.47	0.05	4.42	0.06	4.37	0.07	**0.036**	0.872	0.382

G, generation of rats; P, protection of crops; F, fertilization of crops; TEAC, trolox equivalent antioxidant capacity; TE, trolox equivalent.
*P-values in **bold** are for significant differences (P<0.05); P-values in *italic* are for trends (0.1>P>0.05)

Table S3 continued. Effect of crop protection and fertilization on the daily intake of macronutrients, antioxidants, contaminants and minerals in the first and second generation of rats

		Crop protection (P)				Fertility management (F)				ANOVA results (*P* values*)		
		Organic (- pesticides)		Conventional (+ pesticides)		Organic (composted manure)		Conventional (mineral NPK fertilizer)				
**Feed components**	G	Mean	SEM	Mean	SEM	Mean	SEM	Mean	SEM	P	F	P×F
Contaminants												
Aldicarb (μg)	1	0.000	0.000	0.599	0.088	0.127	0.026	0.462	0.097	**<0.001**	0.188	**<0.001**
	2	0.000	0.000	0.547	0.078	0.139	0.027	0.403	0.088	**<0.001**	0.135	**<0.001**
Diquat (μg)	1	0.000	0.000	0.051	0.007	0.016	0.005	0.034	0.007	**<0.001**	0.370	**0.002**
	2	0.000	0.000	0.049	0.007	0.016	0.004	0.033	0.007	**<0.001**	0.290	**0.001**
Ni (μg)	1	1.39	0.06	1.27	0.05	1.35	0.06	1.32	0.06	0.128	0.751	0.263
	2	1.25	0.06	1.23	0.04	1.27	0.05	1.21	0.05	0.584	0.559	0.697
Cd (μg)	1	0.589	0.027	0.608	0.030	0.422	0.009	0.771	0.015	0.320	**0.006**	0.292
	2	0.549	0.023	0.568	0.029	0.405	0.008	0.705	0.016	**0.036**	**0.006**	**0.016**
Pb (μg)	1	0.686	0.045	0.787	0.057	0.612	0.036	0.857	0.058	*0.085*	0.159	**0.003**
	2	0.628	0.042	0.756	0.057	0.594	0.035	0.786	0.059	**0.026**	0.249	**0.001**
**Minerals**												
Cu (μg)	1	0.047	0.001	0.046	0.001	0.046	0.000	0.048	0.001	0.687	0.585	0.397
	2	0.043	0.001	0.044	0.001	0.044	0.001	0.044	0.001	0.155	0.858	**0.004**

G, generation of rats; P, protection of crops; F, fertilization of crops;
*P-values in **bold** are for significant differences (P<0.05); P-values in *italic* are for trends (0.1>P>0.05)

Table S4. Results of the 3-factor ANOVA of effects of crop protection (P), fertilization (F) and generation (G) on **(a)** growth, basic physiological, endocrine and immunological parameters and **(b)** dietary macronutrient, antioxidant and contaminant intake in rats

	ANOVA results (*P*-values)						
	Main effects			2 and 3-way interactions			
**Parameters assessed**	P	F	G	P×F	P×G	F×G	P×F×G
Growth parameters							
IBW (g)	0.225	0.533	**<0.001**	0.772	**<0.001**	**0.009**	0.321
Feed intake (g/d)	0.203	0.201	**<0.001**	0.722	0.130	**0.013**	*0.099*
Total wt gain (g)	0.870	**0.006**	**<0.001**	0.633	0.562	0.410	0.754
FCR	0.333	*0.098*	**0.013**	0.511	*0.060*	**<0.001**	0.461
Body composition							
%BDM	0.749	0.411	0.971	0.115	0.875	0.609	0.051
Protein (%)	0.705	**0.049**	**<0.001**	0.512	*0.052*	0.189	0.186
Fat (%)	0.538	0.120	0.976	0.439	0.399	0.673	0.750
Ash (%)	0.801	0.557	*0.056*	0.988	0.157	0.938	0.311
Basic physiological parameters							
RBC ($\times 10^6/mm^3$)	0.510	0.310	**<0.001**	*0.070*	0.845	0.895	0.905
Hb (g/100ml)	*0.093*	0.144	**<0.001**	**<0.001**	**0.012**	0.856	**<0.001**
WBC ($\times 10^3/mm^3$)	0.696	0.361	**<0.001**	0.468	0.383	0.268	0.268
PCV (%)	0.902	0.467	**<0.001**	0.191	0.355	0.821	0.591
GLU (mmol/l)	0.122	*0.050*	**<0.001**	0.168	*0.056*	0.758	0.131
TEAC (μmol TE/l)	0.109	0.675	0.329	**0.005**	0.603	0.167	**<0.001**
Plasma hormones							
Cs (ng/ml)	0.128	**0.046**	*0.058*	0.140	**0.002**	0.183	0.693
GH (ng/ml)	0.834	0.689	**0.046**	0.170	0.576	**0.031**	0.422
IGF-1 (μg/ml)	0.612	**0.014**	**<0.001**	**0.011**	**<0.001**	**<0.001**	**<0.001**
Insulin (ng/ml)	*0.098*	0.105	**<0.001**	**0.001**	*0.068*	**0.042**	*0.059*
Leptin (ng/ml)	0.422	0.223	**<0.001**	**0.040**	0.304	0.672	0.357
Ts (ng/ml)	0.755	0.478	**0.046**	*0.065*	0.206	0.603	0.393
Immunoglobulins							
IgA (μg/ml)	**0.037**	0.571	0.815	0.442	**<0.001**	**<0.001**	**<0.001**
IgG (μg/ml)	0.393	0.432	0.744	**0.015**	0.739	**<0.001**	0.788
Immune system responsiveness							
sp-LP ($\times 10^3$ cpm)	0.614	0.219	**0.001**	0.170	**0.004**	**0.023**	**<0.001**
ConA-LP ($\times 10^3$ cpm)	0.616	0.733	**<0.001**	*0.068*	0.869	0.787	**0.037**
LPS-LP ($\times 10^3$ cpm)	**0.028**	0.165	**<0.001**	0.120	0.879	0.467	0.521

P, protection of crops; F, fertilization of crops; G, generation of rats;
*P-values in **bold** are for significant differences (P<0.05); P-values in *italic* are for trends (0.1>P>0.05)
IBW, initial body wt at weaning; FCR, feed conversion ratio calculated as total feed intake/total weight gain over 9 weeks; %BDM, percent of body dry mass; RBC, red blood cells; Hb, hemoglobin; WBC, white blood cells; PCV, blood packed cell volume; GLU, blood glucose; TEAC, trolox equivalent antioxidant capacity; ; TE, trolox equivalent; Cs, corticosterone; GH, growth hormone; IGF-1, insulin-like growth factor 1; Ts, testosterone; IgA, immunoglobulin A; IgG, immunoglobulin G; sp-LP, spontaneous lymphocyte proliferation; LPS-LP, lipopolysaccharide-stimulated lymphocyte proliferation; ConA-LP, concanavalin A-stimulated lymphocyte proliferation.

Table S4 continued. Results (*P*-values) of the 3-factor ANOVA of effects of crop protection, fertilization and generation on **(a)** growth, basic physiological, endocrine and immunological parameters and **(b)** dietary macronutrient, antioxidant and contaminant intake in rats

	ANOVA results (*P*-values)						
	Main effects			2 and 3-way interactions			
**Parameters assessed**	P	F	G	P×F	P×G	F×G	P×F×G
**Macronutrient intake**							
Protein (g)	0.714	**<0.001**	**<0.001**	0.687	*0.084*	**0.008**	*0.082*
Lipid (g)	**0.032**	**0.024**	**<0.001**	0.162	0.137	**0.004**	**0.043**
Fiber (g)	0.260	0.528	**<0.001**	0.553	*0.088*	**0.014**	*0.085*
Ash (g)	0.219	*0.051*	**<0.001**	0.315	0.117	**0.009**	*0.078*
**Antioxidant intake**							
Polyphenols (mg)	**0.002**	**<0.001**	**<0.001**	0.172	**0.045**	*0.066*	0.106
Flavonoids (mg)	0.182	0.123	**<0.001**	0.500	*0.078*	**0.034**	*0.059*
Lutein (mg)	*0.078*	0.265	**<0.001**	0.345	*0.072*	0.158	0.289
β-carotene (mg)	0.944	0.752	**<0.001**	0.645	*0.089*	**0.008**	0.134
TEAC (μmol TE)	0.502	0.537	**<0.001**	0.829	0.132	**0.013**	0.101
Cu (μg)	0.855	0.823	**<0.001**	0.582	0.173	**0.017**	0.163
**Contaminants intake**							
Aldicarb (μg)	*0.071*	0.112	**<0.001**	0.113	**<0.001**	**0.004**	**0.003**
Diquat (μg)	*0.096*	0.300	**0.009**	0.301	**0.009**	0.864	0.863
Cd (μg)	0.656	**<0.001**	**<0.001**	0.568	*0.063*	**<0.001**	*0.072*
Ni (μg)	0.688	0.700	**<0.001**	0.870	0.138	**0.014**	0.192
Pb (μg)	0.510	0.293	**<0.001**	0.327	0.178	*0.074*	0.497

P, protection of crops; F, fertilization of crops; G, generation of rats; TEAC, trolox equivalent antioxidant capacity; TE, trolox equivalent.

Table S5. Rat body composition and basic physiological parameters in the first and second generation of rats.

Parameter Assessed	G	Crop protection (P)				Fertility management (F)				ANOVA results (*P* values*)		
		Organic (- pesticides)		Conventional (+ pesticides)		Organic (composted manure)		Conventional (mineral NPK fertilizer)				
		Mean	SEM	Mean	SEM	Mean	SEM	Mean	SEM	P	F	P×F
**Body composition**												
Protein (%)	1	21.6	0.1	21.5	0.1	21.3	0.1	21.9	0.1	0.327	*0.060*	0.722
	2	22.2	0.1	22.5	0.1	22.2	0.1	22.4	0.1	0.151	0.386	0.180
Fat (%)	1	9.81	0.32	9.55	0.30	10.15	0.30	9.18	0.30	0.601	0.268	0.407
	2	9.90	0.30	9.41	0.24	10.05	0.28	9.28	0.26	0.133	0.238	0.128
Ash (%)	1	3.57	0.04	3.50	0.04	3.52	0.04	3.55	0.04	0.228	0.614	0.421
	2	3.60	0.05	3.64	0.04	3.60	0.04	3.63	0.04	0.479	0.704	0.526
%BDM	1	37.3	0.3	37.0	0.3	37.4	0.3	36.9	0.3	0.430	0.463	0.267
	2	37.2	0.3	37.0	0.3	37.2	0.3	37.0	0.3	0.714	0.686	**<0.001**
**Basic physiological parameters**												
RBC ($\times10^6/mm^3$)	1	10.0	0.2	10.2	0.2	10.2	0.2	10.0	0.2	0.593	0.532	0.233
	2	8.84	0.13	8.95	0.09	8.98	0.11	8.82	0.12	0.501	0.409	**0.017**
WBC ($\times10^3/mm^3$)	1	7.00	0.39	7.42	0.41	7.56	0.45	6.88	0.35	0.462	0.303	0.850
	2	11.4	0.2	11.2	0.2	11.3	0.3	11.3	0.2	0.689	0.899	*0.068*
Hb (g/100ml)	1	15.2	0.2	15.2	0.2	15.4	0.1	15.0	0.2	0.945	0.269	0.340
	2	17.1	0.4	16.0	0.3	16.7	0.4	16.3	0.3	**0.003**	0.315	**<0.001**
PCV (%)	1	51.1	0.6	51.4	0.7	51.5	0.7	51.0	0.6	0.663	0.709	0.237
	2	46.6	0.4	46.0	0.4	46.4	0.4	46.2	0.4	0.312	0.625	0.420
GLU (mmol/l)	1	7.40	0.23	7.22	0.24	7.02	0.18	7.60	0.27	0.613	0.304	0.832
	2	9.26	0.42	7.92	0.30	8.13	0.37	8.96	0.37	**0.009**	0.361	*0.050*
TEAC (μmol TE)	1	2.83	0.03	2.76	0.03	2.81	0.02	2.77	0.03	**0.011**	0.306	**<0.001**
	2	2.84	0.01	2.79	0.02	2.80	0.01	2.82	0.02	**0.023**	0.560	0.565

G, generation of rats; P, protection of crops; F, fertilization of crops; %BDM, percent of body dry mass; RBC, red blood cells; WBC, white blood cells; PCV, blood packed cell volume; Hb, hemoglobin; GLU, blood glucose; TEAC, trolox equivalent antioxidant capacity; TE, trolox equivalent.
*P-values in **bold** are for significant differences (P<0.05); P-values in *italic* are for trends (0.1>P>0.05)

Table S6. Effect of crop protection and fertilization on plasma hormones, immunoglobulin concentrations and immune system responsiveness in the first and second generation of rats

		Crop protection (P)				Fertility management (F)						
		Organic (- pesticides)		Conventional (+ pesticides)		Organic (composted manure)		Conventional (mineral NPK fertilizer)		ANOVA results (*P* values*)		
	G	Mean	SEM	Mean	SEM	Mean	SEM	Mean	SEM	P	F	P×F
Plasma hormones												
Cs (ng/ml)	1	363	21	490	30	446	24	405	30	**0.001**	0.337	0.246
	2	385	25	378	20	436	26	329	15	0.737	*0.058*	**0.041**
GH (ng/ml)	1	28.8	3.6	29.3	3.4	33.2	4.2	25.5	2.7	0.928	0.354	0.519
	2	24.8	2.6	22.1	2.9	21.0	2.6	25.8	2.9	0.481	0.290	**0.038**
IGF-1 (μg/ml)	1	1.62	0.05	1.83	0.04	1.72	0.04	1.71	0.05	**0.001**	0.856	**0.026**
	2	1.63	0.09	1.35	0.05	1.29	0.05	1.69	0.08	**<0.001**	**0.018**	**<0.001**
Insulin (ng/ml)	1	1.73	0.16	2.43	0.26	2.38	0.26	1.77	0.16	**0.009**	*0.090*	**<0.001**
	2	1.36	0.15	1.47	0.13	1.43	0.14	1.41	0.14	0.595	0.891	**<0.001**
Leptin (ng/ml)	1	4.69	0.40	3.94	0.34	4.67	0.39	4.04	0.37	0.152	0.448	0.256
	2	3.20	0.28	3.15	0.24	3.37	0.26	2.98	0.26	0.871	0.382	**0.001**
Ts (ng/ml)	1	0.92	0.09	0.85	0.11	0.88	0.09	0.90	0.11	0.627	0.832	0.345
	2	0.65	0.07	0.79	0.06	0.66	0.06	0.78	0.07	0.109	0.421	**0.002**
Immunoglobulins												
IgA (μg/ml)	1	79	5	140	5	95	8	123	5	**<0.001**	*0.068*	**<0.001**
	2	116	5	105	4	121	5	100	4	**0.032**	**0.026**	**<0.001**
IgG (μg/ml)	1	71	1	69	1	73	1	67	1	**0.005**	**0.027**	**<0.001**
	2	71	1	69	1	68	1	71	2	*0.055*	*0.700*	**<0.001**
Immune system responsiveness												
sp-LP (×10^3 cpm)	1	10.6	0.7	11.7	1.0	11.1	0.9	11.2	0.9	0.389	0.950	**0.008**
	2	10.1	2.3	4.6	0.7	9.9	2.3	4.8	0.7	**0.009**	0.175	**<0.001**
ConA-LP (×10^3 cpm)	1	86.9	6.9	83.3	7.6	85.7	7.2	84.5	7.4	0.725	0.919	0.660
	2	52.3	6.7	46.4	7.3	51.8	7.0	46.9	7.0	0.527	0.633	**<0.001**
LPS-LP (×10^3 cpm)	1	10.02	1.06	6.18	1.49	9.44	1.36	6.77	1.25	**0.032**	0.230	0.485
	2	5.53	1.29	1.27	0.34	3.94	1.14	2.48	0.52	**<0.001**	0.489	0.020

G, generation of rats; P, protection of crops; F, fertilization of crops; Cs, corticosterone; GH, growth hormone; IGF-1, insulin-like growth factor 1; Ts, testosterone; IgA, immunoglobulin A; IgG, immunoglobulin G; sp-LP, spontaneous lymphocyte proliferation; LPS-LP, lipopolysaccharide-stimulated lymphocyte proliferation; ConA-LP, concanavalin A-stimulated lymphocyte proliferation.

*P-values in **bold** are for significant differences (P<0.05); P-values in *italic* are for trends (0.1>P>0.05)

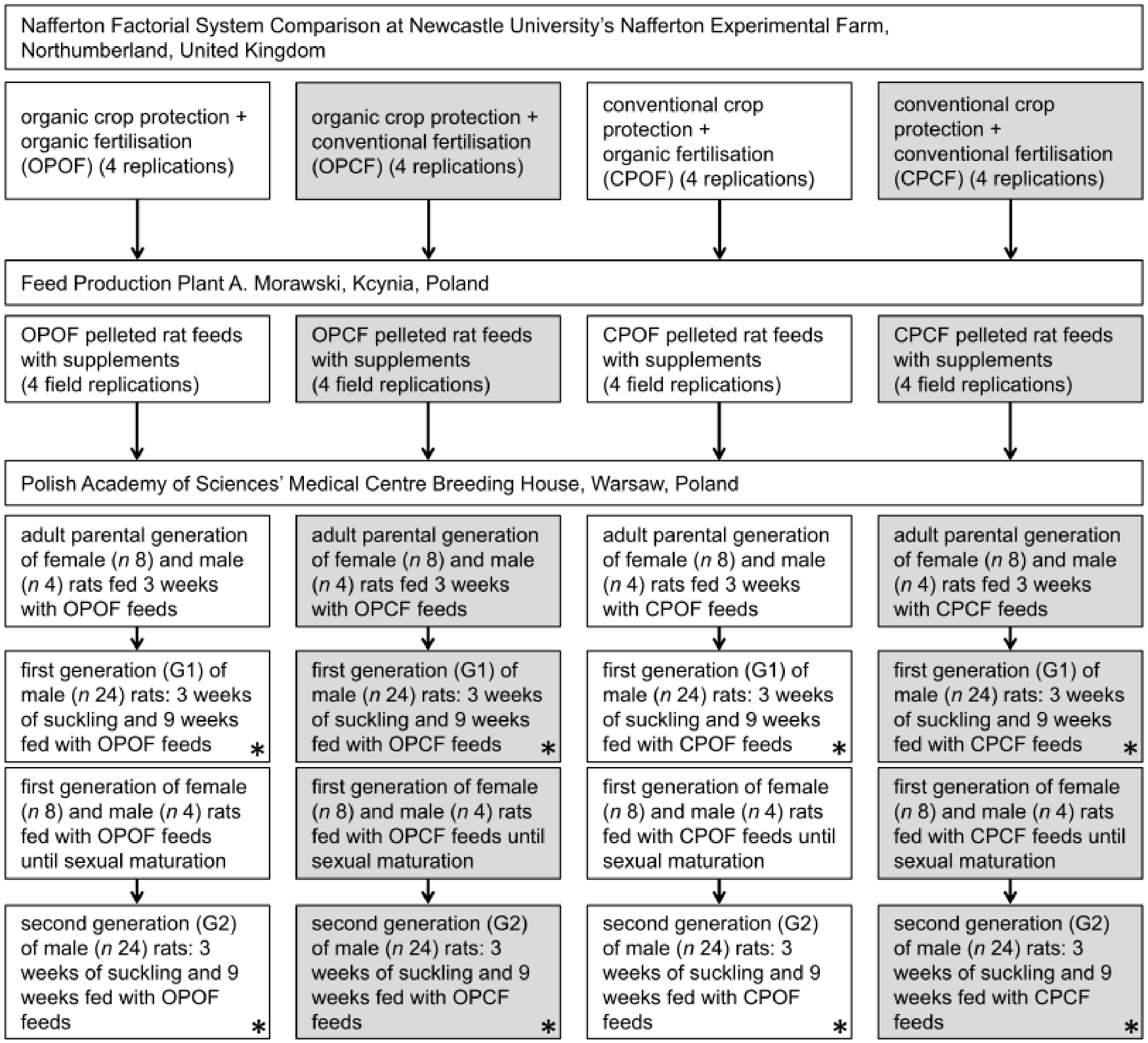

Figure S1 Experimental design flow diagram. *animals assessed in trials

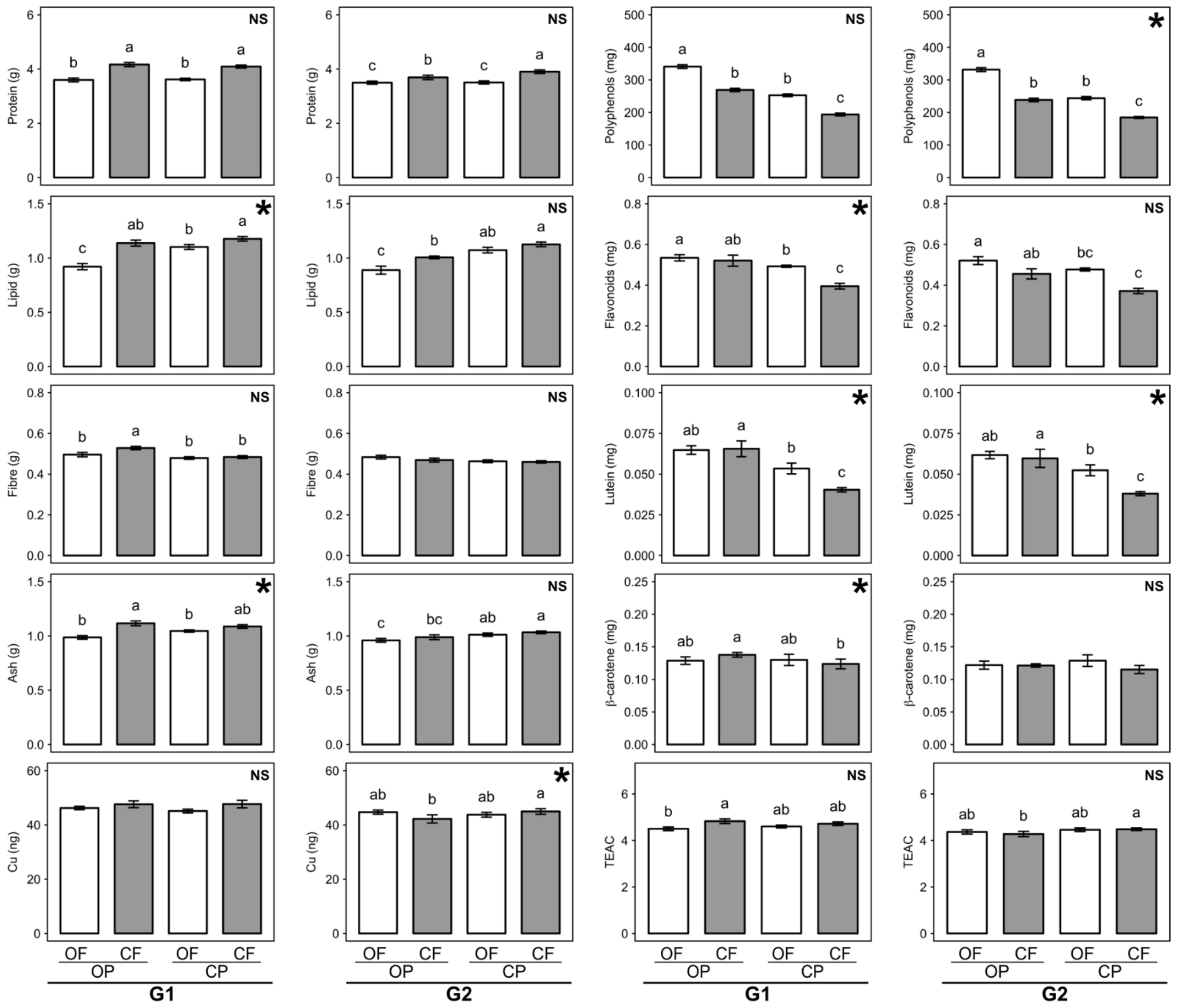

Figure S2. Effects of organic crop protection (OP) or conventional crop protection (CP) and organic fertility management (OF) or conventional fertility management (CF) on the daily intake of protein, lipid, fiber, ash, Cu, polyphenols, flavonoids, lutein, β-carotene, antioxidant capacity (TEAC) of the first (G1) and second (G2) generation of rats. Results shown as means ±SEM of n = 24 animals, different letters above bars indicate significant difference ($P \leq 0.05$) determined by Tukey's HSD test. * two factor ANOVA detected a significant interaction between crop protection and fertilization regimes (see Table S3 for ANOVA *P*-values and main effect means).

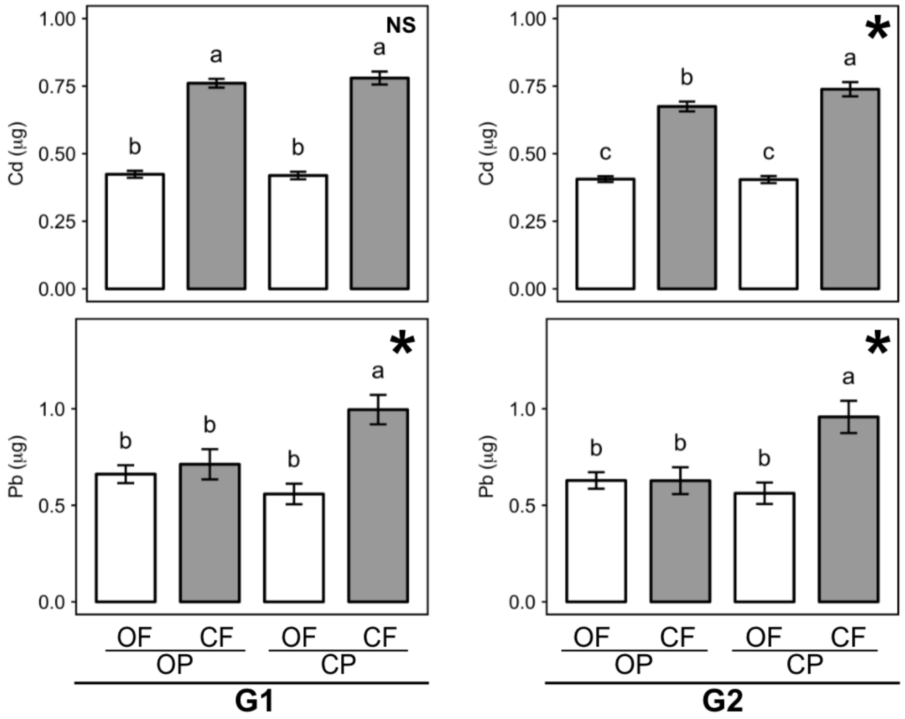

Figure S3. Effects of organic crop protection (OP) or conventional crop protection (CP) and organic fertility management (OF) or conventional fertility management (CF) on the daily intake of cadmium (Cd) and lead (Pb) of the first (G1) and second (G2) generation of rats. Results shown as means ±SEM of n = 24 animals, different letters above bars indicate significant difference ($P \leq 0.05$) determined by Tukey's HSD test. * two factor ANOVA detected a significant interaction between crop protection and fertilization regimes (see Table S3 for ANOVA *P*-values and main effect means).

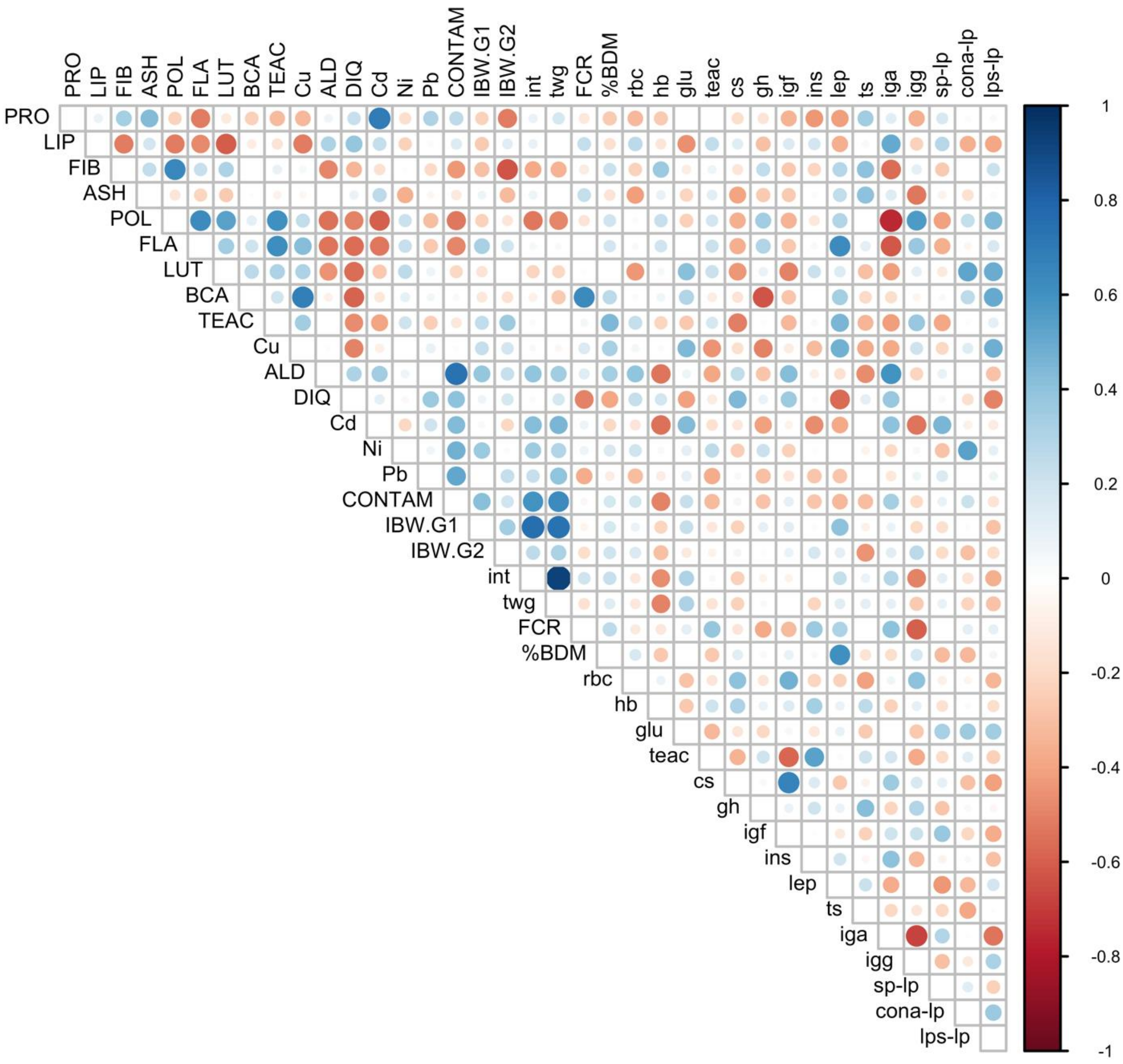

Figure S4. Correlation between concentration of experimental feed components and physiological parameters for the first generation of rats (G1). The Pearson correlation was indicated on the color scale (blue as positive and red as negative), where color intensity and circle sizes are proportional to the correlation coefficient. PRO, protein; LIP, lipids; FIB, fiber; ASH, ash; POL, polyphenols; FLA, flavonols; LUT, lutein; BCA, β-carotene; TEAC, feed antioxidant activity; Cu, copper; ALD, aldicarb; DIQ, diquat; Cd, cadmium; Ni, nickel; Pb, lead; CONTAM, contaminants altogether; IBW.G1, initial body weight of first generation of rats; IBW.G2, initial body weight of second generation of rats; int, total feed intake; twg, total weight gain; FCR, feed conversion ratio; %BDM, percent of body dry matter; rbc, red blood cells count; hb, blood hemoglobin content; glu, plasma glucose content; teac, plasma antioxidant capacity; cs, corticosterone; gh, growth hormone; igf, insulin-like growth factor 1; ins, insulin; lep, leptin; ts, testosterone; iga, immunoglobulin A; igg, immunoglobulin G; sp-lp, spontaneous lymphocyte proliferation; cona-lp, concanavalin A-stimulated lymphocyte proliferation; lps-lp, lipopolysaccharide-stimulated lymphocyte proliferation.

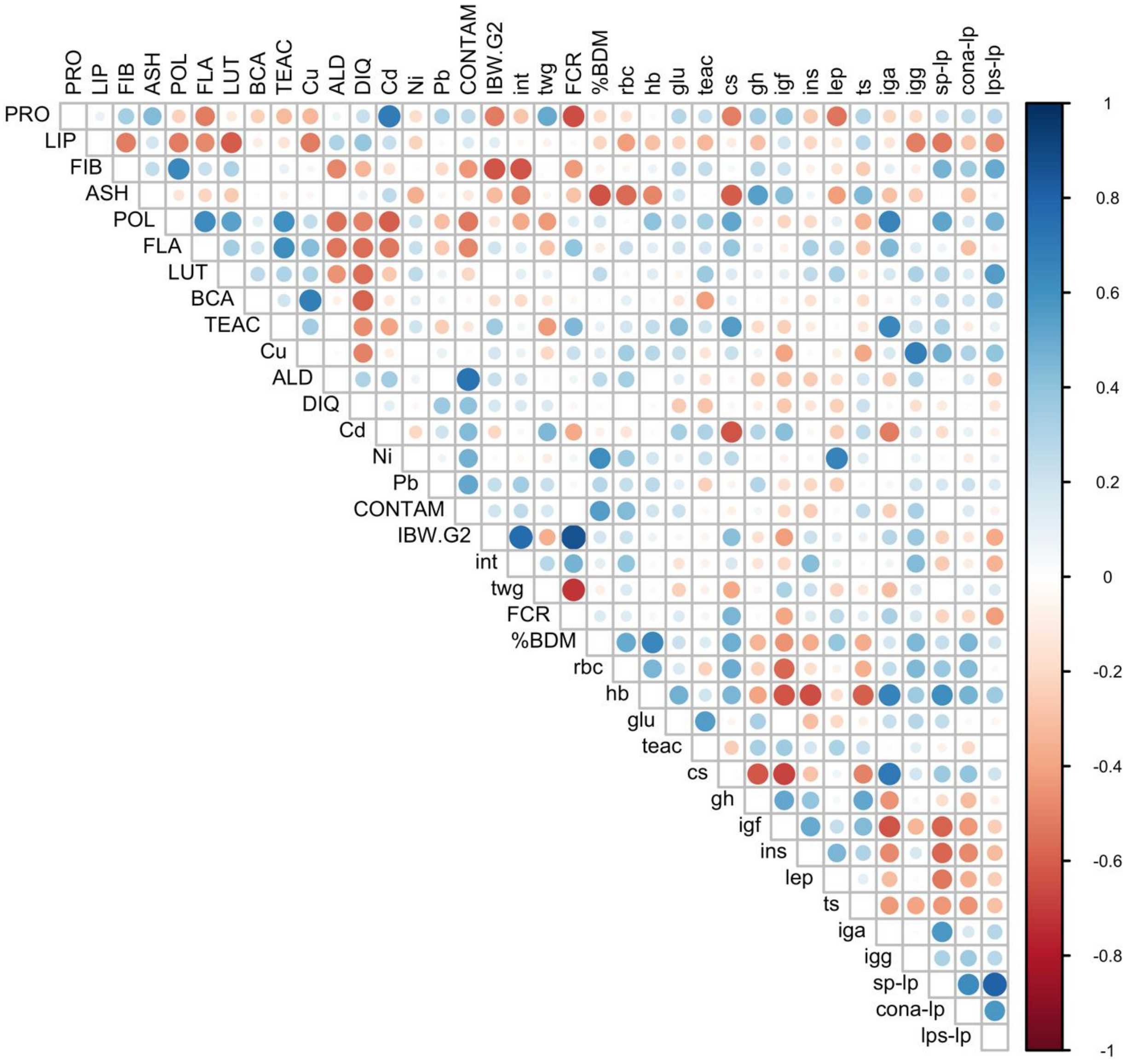

Figure S5. Correlation between concentration of experimental feed components and physiological parameters for the second generation of rats (G2). The Pearson correlation was indicated on the color scale (blue as positive and red as negative), where color intensity and circle sizes are proportional to the correlation coefficient. PRO, protein; LIP, lipids; FIB, fiber; ASH, ash; POL, polyphenols; FLA, flavonols; LUT, lutein; BCA, β-carotene; TEAC, feed antioxidant activity; Cu, copper; ALD, aldicarb; DIQ, diquat; Cd, cadmium; Ni, nickel; Pb, lead; CONTAM, contaminants altogether; IBW.G2, initial body weight of second generation of rats; int, total feed intake; twg, total weight gain; FCR, feed conversion ratio; %BDM, percent of body dry matter; rbc, red blood cells count; hb, blood hemoglobin content; glu, plasma glucose content; teac, plasma antioxidant capacity; cs, corticosterone; gh, growth hormone; igf, insulin-like growth factor 1; ins, insulin; lep, leptin; ts, testosterone; iga, immunoglobulin A; igg, immunoglobulin G; sp-lp, spontaneous lymphocyte proliferation; cona-lp, concanavalin A-stimulated lymphocyte proliferation; lps-lp, lipopolysaccharide-stimulated lymphocyte proliferation.

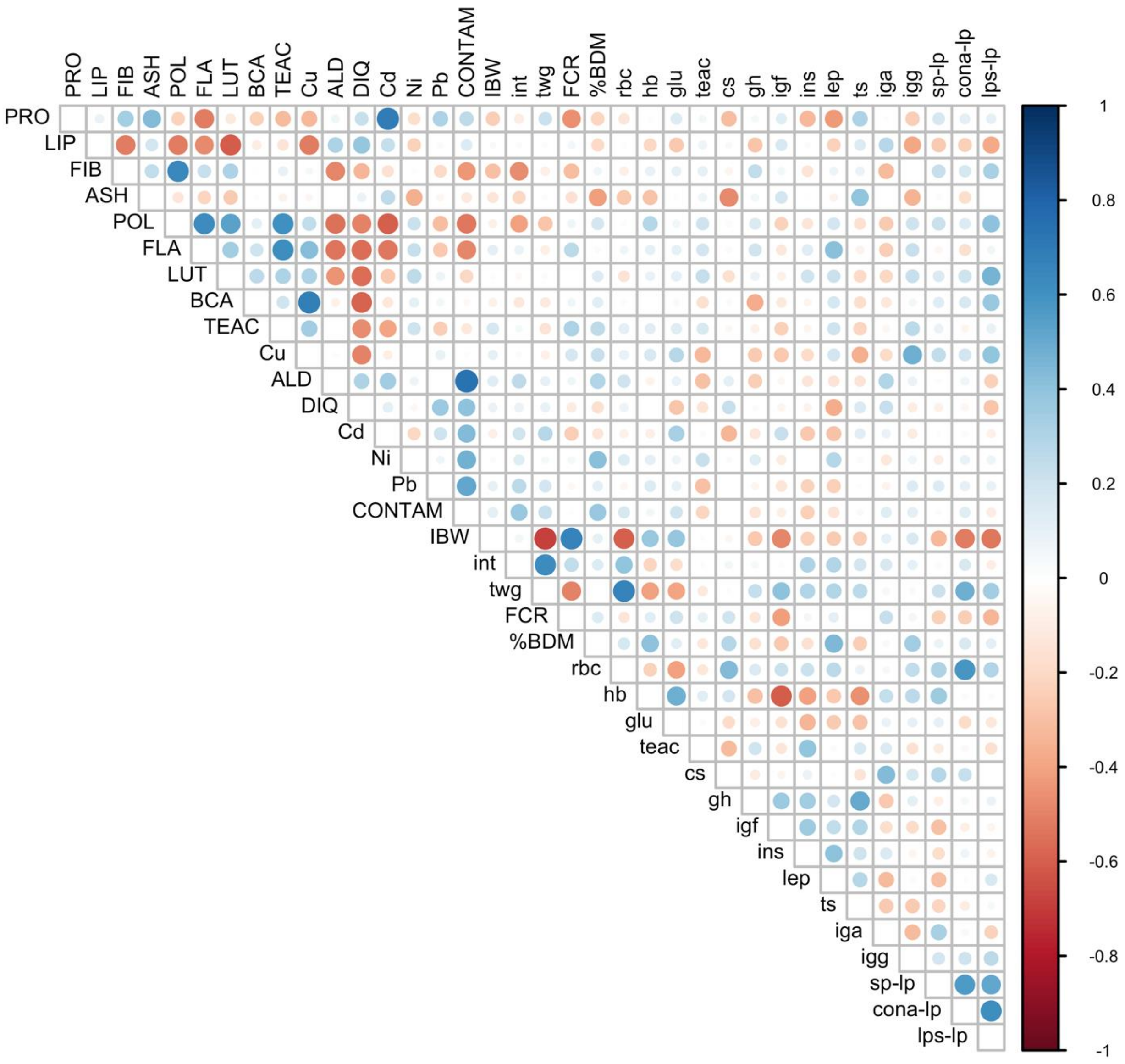

Figure S6. Correlation between concentration of experimental feed components and physiological parameters for both generations of rats (G1+G2). The Pearson correlation was indicated on the color scale (blue as positive and red as negative), where color intensity and circle sizes are proportional to the correlation coefficient. PRO, protein; LIP, lipids; FIB, fiber; ASH, ash; POL, polyphenols; FLA, flavonols; LUT, lutein; BCA, β-carotene; TEAC, feed antioxidant activity; Cu, copper; ALD, aldicarb; DIQ, diquat; Cd, cadmium; Ni, nickel; Pb, lead; CONTAM, contaminants altogether; IBW.G1, initial body weight of first generation of rats; IBW, initial body weight of rats; int, total feed intake; twg, total weight gain; FCR, feed conversion ratio; %BDM, percent of body dry matter; rbc, red blood cells count; hb, blood hemoglobin content; glu, plasma glucose content; teac, plasma antioxidant capacity; cs, corticosterone; gh, growth hormone; igf, insulin-like growth factor 1; ins, insulin; lep, leptin; ts, testosterone; iga, immunoglobulin A; igg, immunoglobulin G; sp-lp, spontaneous lymphocyte proliferation; cona-lp, concanavalin A-stimulated lymphocyte proliferation; lps-lp, lipopolysaccharide-stimulated lymphocyte proliferation.

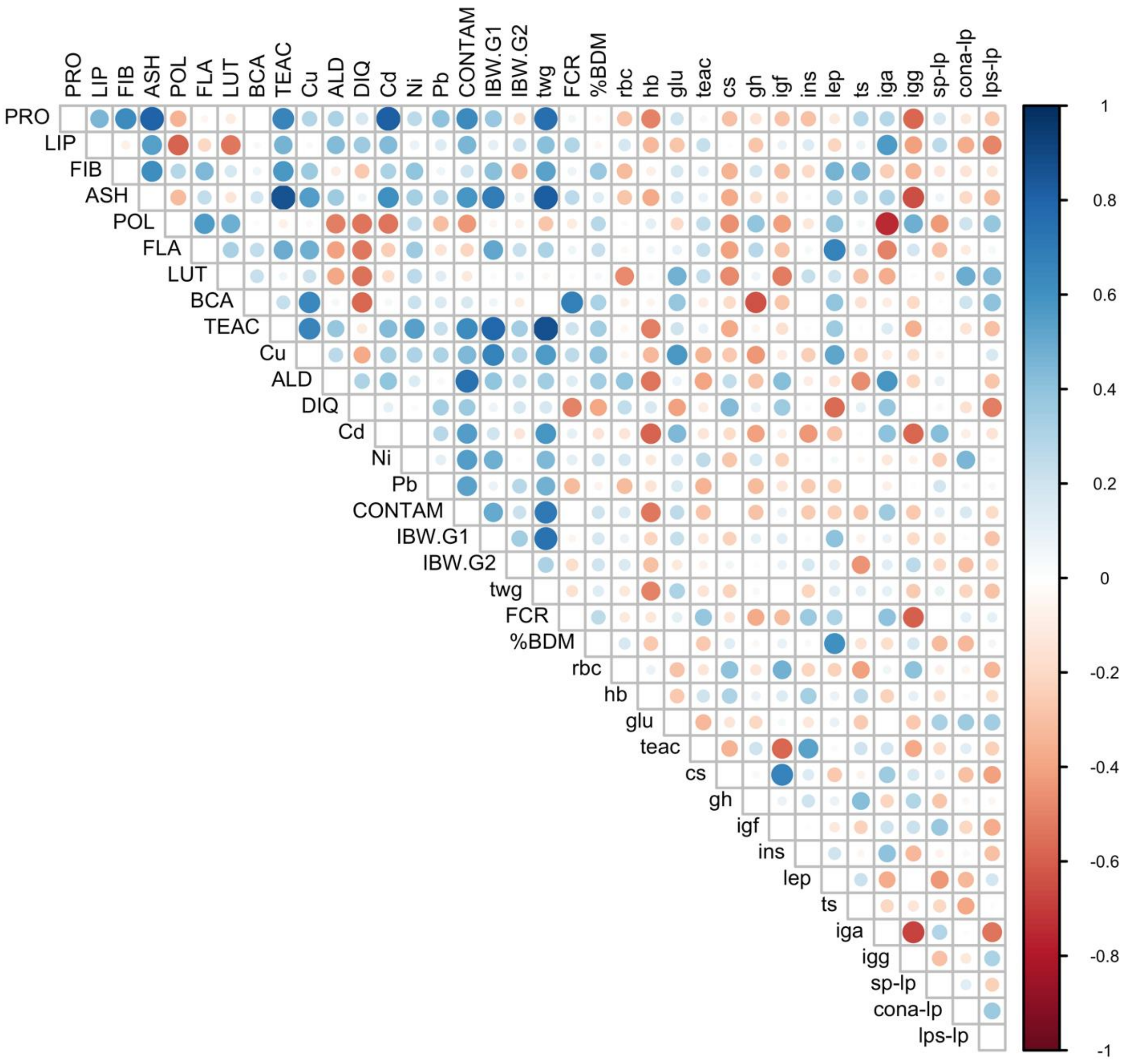

Figure S7. Correlation between calculated intake of experimental feed components and physiological parameters for the first generation of rats (G1). The Pearson correlation was indicated on the color scale (blue as positive and red as negative), where color intensity and circle sizes are proportional to the correlation coefficient. PRO, protein; LIP, lipids; FIB, fiber; ASH, ash; POL, polyphenols; FLA, flavonols; LUT, lutein; BCA, β-carotene; TEAC, feed antioxidant activity; Cu, copper; ALD, aldicarb; DIQ, diquat; Cd, cadmium; Ni, nickel; Pb, lead; CONTAM, contaminants altogether; IBW.G1, initial body weight of first generation of rats; IBW.G2, initial body weight of second generation of rats; int, total feed intake; twg, total weight gain; FCR, feed conversion ratio; %BDM, percent of body dry matter; rbc, red blood cells count; hb, blood hemoglobin content; glu, plasma glucose content; teac, plasma antioxidant capacity; cs, corticosterone; gh, growth hormone; igf, insulin-like growth factor 1; ins, insulin; lep, leptin; ts, testosterone; iga, immunoglobulin A; igg, immunoglobulin G; sp-lp, spontaneous lymphocyte proliferation; cona-lp, concanavalin A-stimulated lymphocyte proliferation; lps-lp, lipopolysaccharide-stimulated lymphocyte proliferation.

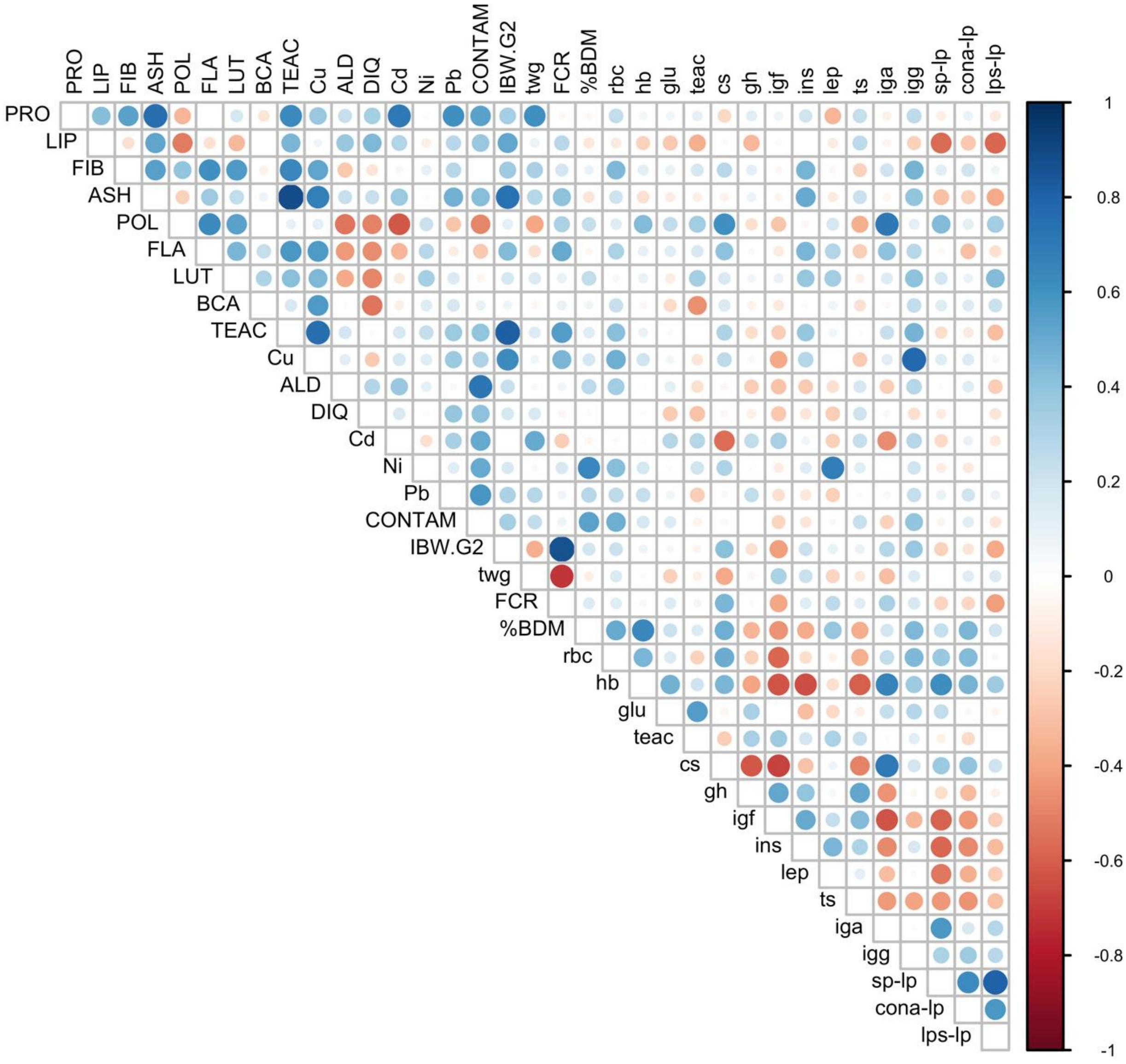

Figure S8. Correlation between calculated intake of experimental feed components and physiological parameters for the second generation of rats (G2). The Pearson correlation was indicated on the color scale (blue as positive and red as negative), where color intensity and circle sizes are proportional to the correlation coefficient. PRO, protein; LIP, lipids; FIB, fiber; ASH, ash; POL, polyphenols; FLA, flavonols; LUT, lutein; BCA, β-carotene; TEAC, feed antioxidant activity; Cu, copper; ALD, aldicarb; DIQ, diquat; Cd, cadmium; Ni, nickel; Pb, lead; CONTAM, contaminants altogether; IBW.G2, initial body weight of second generation of rats; int, total feed intake; twg, total weight gain; FCR, feed conversion ratio; %BDM, percent of body dry matter; rbc, red blood cells count; hb, blood hemoglobin content; glu, plasma glucose content; teac, plasma antioxidant capacity; cs, corticosterone; gh, growth hormone; igf, insulin-like growth factor 1; ins, insulin; lep, leptin; ts, testosterone; iga, immunoglobulin A; igg, immunoglobulin G; sp-lp, spontaneous lymphocyte proliferation; cona-lp, concanavalin A-stimulated lymphocyte proliferation; lps-lp, lipopolysaccharide-stimulated lymphocyte proliferation.

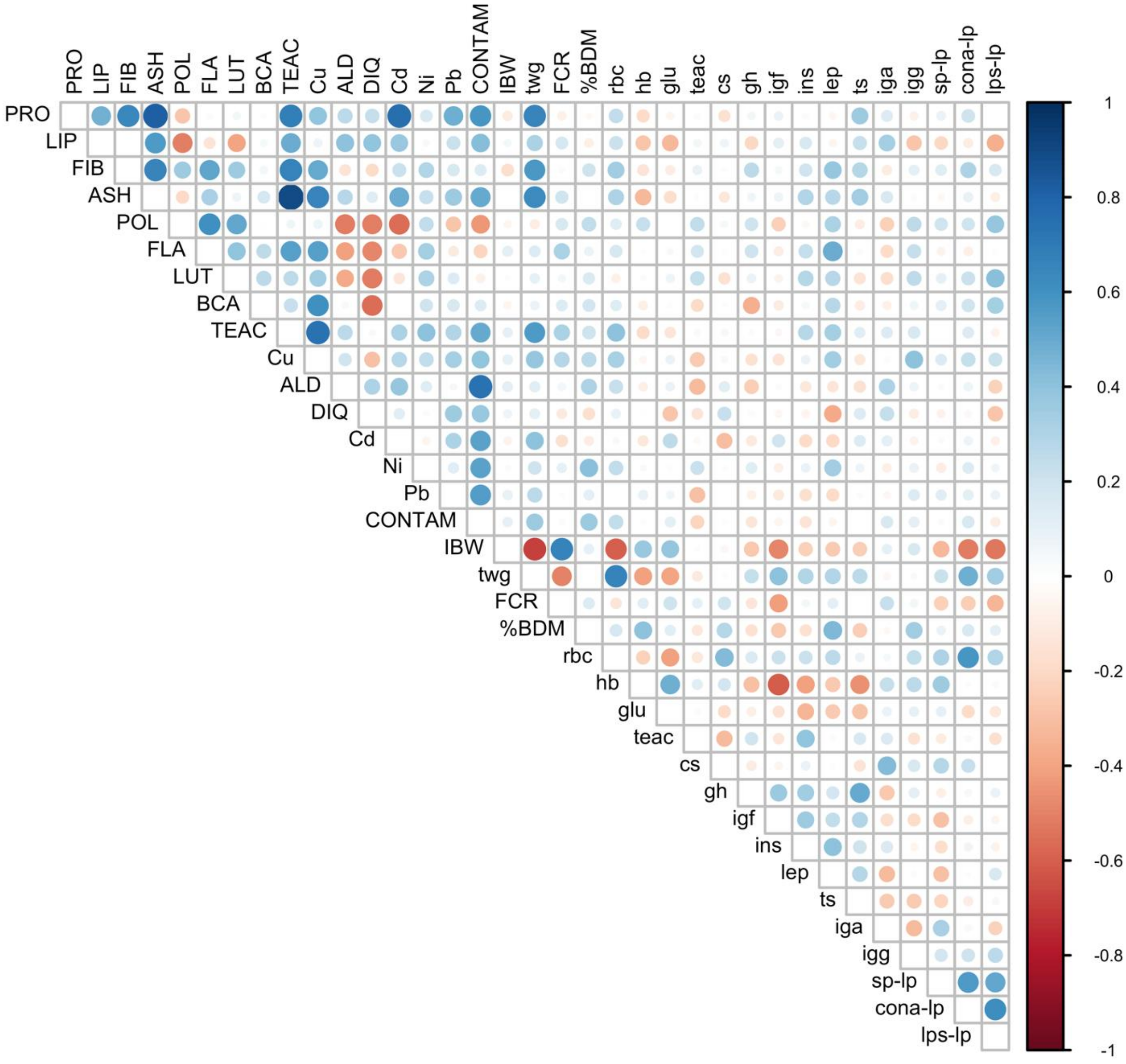

Figure S9. Correlation between calculated intake of experimental feed components and physiological parameters for both generations of rats (G1+G2). The Pearson correlation was indicated on the color scale (blue as positive and red as negative), where color intensity and circle sizes are proportional to the correlation coefficient. PRO, protein; LIP, lipids; FIB, fiber; ASH, ash; POL, polyphenols; FLA, flavonols; LUT, lutein; BCA, β-carotene; TEAC, feed antioxidant activity; Cu, copper; ALD, aldicarb; DIQ, diquat; Cd, cadmium; Ni, nickel; Pb, lead; CONTAM, contaminants altogether; IBW.G1, initial body weight of first generation of rats; IBW, initial body weight of rats; int, total feed intake; twg, total weight gain; FCR, feed conversion ratio; %BDM, percent of body dry matter; rbc, red blood cells count; hb, blood hemoglobin content; glu, plasma glucose content; teac, plasma antioxidant capacity; cs, corticosterone; gh, growth hormone; igf, insulin-like growth factor 1; ins, insulin; lep, leptin; ts, testosterone; iga, immunoglobulin A; igg, immunoglobulin G; sp-lp, spontaneous lymphocyte proliferation; cona-lp, concanavalin A-stimulated lymphocyte proliferation; lps-lp, lipopolysaccharide-stimulated lymphocyte proliferation.
